# Hereditary Breast Cancer in a Brazilian Referral Cohort: Germline Diversity and Genotype–Phenotype Associations

**DOI:** 10.3390/ijms27219686

**Published:** 2026-10-29

**Authors:** André Luiz Cicilini, Patrick Vinicius Cerqueira Batista, Sandrine M. Caputo, Viviane Primo Basilio de Souza, Solange Moraes Sanchez, Fabiana Baroni Makdissi, Marina de Brot, Marina de Assis da Escossia Fernandes, Beatriz Nelli Barbatto, Leticia Coelho de Mattos, Nathalia Machado Soldi, Flavia Cavalcanti Ballint, Débora Guilherme de Albuquerque e Rodrigues de Sousa, Eduarda Mirella da Silva Montiel, Lurick Rodrigues Soares, Thamilyn Yoshizaki Saruwatari, Sahlua Miguel Volc, Solange Maria Torchia Carvalho Castro, Giovana Tardin Torrezan, Dirce Maria Carraro, Alexandre Andre Balliero, Vladmir Cláudio Cordeiro de Lima, José Claudio Casali-da-Rocha, Elizabeth Santana dos Santos

**Affiliations:** 1 Department of Medical Oncology, A.C. Camargo Cancer Center, São Paulo 01509-010, Brazil; andre.cicilini@accamargo.org.br (A.L.C.);; 2 Department of Genetics, Institut Curie, Paris Sciences and Lettres (PSL) University, 75005 Paris, France; sandrine.caputo@curie.fr; 3 Breast Cancer Reference Center , A.C. Camargo Cancer Center, São Paulo 01509-010, Brazil; 4 Department of Pathological Anatomy, A.C. Camargo Cancer Center, São Paulo 01509-010, Brazil; marina.debrot@accamargo.org.br; 5 Department of Oncogenetics, A.C. Camargo Cancer Center, São Paulo 01509-010, Brazil; 6 Department of Molecular Biology and Genetics, International Research Center (CIPE), A.C. Camargo Cancer Center, São Paulo 01509-010, Brazil; 7 Department of Oncogenetics, Hospital Sírio Libanês, São Paulo 01308-060, Brazil

**Keywords:** hereditary breast cancer, germline pathogenic variants, genetic diversity, admixed populations, genotype–phenotype associations, genetic testing access

## Abstract

The germline landscape of hereditary breast cancer has been characterized predominantly in populations of European ancestry, with limited evidence from populations with different demographic histories. We investigated germline diversity and genotype–phenotype associations in a single-center ambispective cohort at a tertiary referral cancer center in Brazil. Among 10,098 individuals who underwent germline genetic testing, 503 adults with invasive or microinvasive breast cancer met the predefined eligibility criteria and carried 514 pathogenic or likely pathogenic germline variants. Analyses assessed gene distribution, recurrent and founder variants, ecological diversity metrics, and associations with clinicopathological characteristics and survival outcomes. *BRCA1* (31.6%) and *BRCA2* (21.7%) were the most frequently altered susceptibility genes, followed by *TP53* (13.1%), including the Brazilian founder variant *TP53* p.R337H (10.7%). Diversity analyses indicated substantial heterogeneity beyond *BRCA1/2*. In exploratory unadjusted analyses, *BRCA1*-associated tumors more frequently exhibited triple-negative phenotype, high histological and nuclear grade, elevated Ki-67, and increased tumor-infiltrating lymphocytes, whereas tumors associated with *BRCA2, ATM, CHEK2,* and mismatch repair genes more frequently exhibited luminal phenotypes. Recurrent variants included alleles previously reported in different geographic and population contexts. Exploratory survival analyses detected no statistically significant differences among genetic groups within a five-year analytical horizon. These findings describe a heterogeneous germline spectrum and exploratory genotype–phenotype associations in a Brazilian cohort. Heterogeneous genetic testing coverage and the absence of individual genomic ancestry assessment limit interpretation; the findings should not be considered ancestry-specific or nationally representative. Further studies with uniform genetic testing and genomic ancestry assessment are warranted in broader Brazilian and Latin American cohorts.

## 1. Introduction

Hereditary breast cancer accounts for 5–10% of all breast cancer (BC) cases and is classically associated with *BRCA1* and *BRCA2*-related hereditary breast and ovarian cancer syndrome (HBOC). *BRCA1/2* explain nearly half of familial aggregation and are associated with early-onset disease [1]. The introduction of next-generation sequencing (NGS) has expanded the spectrum of susceptibility genes beyond *BRCA1/2* [2], encompassing high-penetrance genes such as *TP53*, *PALB2*, *PTEN*, and *CDH1*, together with moderate-penetrance DNA damage repair genes including *ATM*, *CHEK2*, *RAD51C/D*, *BARD1*, and *BRIP1*, whose penetrance varies across populations [3,4,5,6,7,8,9,10].

Because the frequency and spectrum of pathogenic or likely pathogenic (P/LP) variants differ across populations, region-specific evidence is essential to inform hereditary cancer testing. This need is particularly relevant in Latin America, where populations have been shaped by complex demographic histories and extensive population-level admixture [11,12,13,14]. Brazil exemplifies this complexity, with centuries of admixture involving Indigenous American, European, African, and other ancestral contributions [11,12,15,16,17]. However, population-level admixture, self-reported racial or ethnic categories, and individual genomic ancestry are related but non-equivalent concepts. Individual genomic ancestry may vary substantially among persons within the same self-reported racial or ethnic category, making self-identification an imperfect proxy for genomic ancestry [11,12].

Evidence suggests that genomic ancestry may be associated with the frequency and variant spectrum of hereditary breast cancer susceptibility genes, including *BRCA1/2*, *TP53*, *CHEK2*, and homologous recombination repair genes [13,14,16,18,19,20]. Germline genotype may also be associated with tumor phenotype, proliferative activity, immune microenvironment, and recurrence patterns [21,22,23]. However, most genotype–phenotype evidence derives from populations of predominantly European ancestry [7,24], and integrated analyses combining germline findings with detailed clinicopathological characteristics remain limited in Latin American cohorts [4,16,17,25].

Access to comprehensive genetic testing is heterogeneous in Brazil and other Latin American settings [2,4,8,12,16,17,18,25]. Restricted testing may fail to identify clinically relevant variants outside *BRCA1/2*, whereas broader multigene testing introduces additional costs and interpretative complexity. Testing pathways and gene-level coverage may therefore influence the germline spectrum observed in clinically ascertained cohorts. Quantitative approaches adapted from ecology, including diversity indices and Hill numbers, provide a framework for describing observed germline heterogeneity beyond simple variant counts [26,27].

Because individual genomic ancestry was not determined, ancestry-specific associations could not be evaluated. Accordingly, this study aimed to characterize the observed spectrum and diversity of germline P/LP variants and to evaluate genotype–phenotype associations in a Brazilian hereditary breast cancer referral cohort. These analyses were intended to describe this clinically ascertained single-center cohort and not to generate ancestry-specific associations or nationally representative prevalence estimates.

## 2. Results

### 2.1. Clinical and Epidemiological Characteristics

Between January 2019 and July 2025, 10,098 individuals underwent germline genetic testing at our institution, of whom 503 met the eligibility criteria and comprised the final analytic cohort. The cohort included predominantly women (*n* = 501, 99.6%), with a median age at breast cancer diagnosis of 42 years (IQR 36–49; Table 1). Age at diagnosis differed across germline genetic groups (Kruskal–Wallis χ^2^ = 23.05; df = 8; *p* = 0.003), ranging from a median of 40 years (IQR 35–50) among *TP53* carriers to 49 years (IQR 39–59) among *RAD* family carriers (Supplementary Table S1). After Bonferroni correction, only the comparison between *BRCA1* and the rare-gene group remained significant (adjusted *p* = 0.020), with *BRCA1* carriers diagnosed a median of 6 years earlier (Hodges–Lehmann estimate −6 years, 95% CI −10 to −3).

The distribution of P/LP variants across gene groups did not differ significantly between White and Afro-descendant participants (Fisher’s exact test with Monte Carlo simulation, *p* = 0.115). No ethnic group–gene group combination showed a significant deviation from the expected distribution (all absolute adjusted standardized residuals < 1.96).

A family history of cancer was reported by 354 patients (70.4%), whereas 149 (29.6%) reported no family history or were adopted (Table 1). Synchronous tumors were evaluated across genetic groups; the *TP53* group showed the broadest tumor spectrum, whereas tumors observed among *BRCA1*, *BRCA2*, and mismatch repair (MMR) variant carriers were consistent with the recognized tumor spectra of the corresponding hereditary cancer syndromes. Additional findings are provided in Supplementary Table S2 and Figure 1.

Most patients presented with early-stage disease: 399 (79.8%) had T1–T2 tumors, 307 (61.6%) had no nodal involvement, and 397 (79.4%) had stage I–II disease. De novo metastatic disease was uncommon (*n* = 9, 1.8%). Clinical stage distribution did not differ significantly across genetic groups (Fisher’s exact test with Monte Carlo simulation, *p* = 0.43).

### 2.2. Germline Landscape and Genetic Diversity

Among 10,098 individuals who underwent germline genetic testing during the study period, 503 met the eligibility criteria and comprised the final analytic cohort. Overall, 514 P/LP variants were identified across 30 genes. Their distribution is shown in Figure 2, with detailed gene-level data provided in Supplementary Tables S3 and S4.

P/LP variants were most frequently identified in *BRCA1* (*n* = 159, 31.6%), *BRCA2* (*n* = 109, 21.7%), and *TP53* (*n* = 66, 13.1%). The Brazilian founder variant *TP53* c.1010G>A (p.R337H; rs121912651) accounted for 81% of all *TP53* P/LP variants. Moderate-penetrance genes, including *ATM* (*n* = 33, 6.6%), *PALB2* (*n* = 30, 6.0%), and CHEK2 (*n* = 29, 5.8%), collectively accounted for a substantial proportion of the cohort. *RAD* family genes (*n* = 22, 4.4%) and mismatch repair (MMR) genes (*n* = 12, 2.4%) accounted for smaller proportions.

Ten patients (2.0%) carried multiple germline P/LP variants and were classified as having multilocus inherited neoplasia allele syndrome (MINAS). Nine patients carried two P/LP variants, whereas one patient carried three, corresponding to 21 P/LP variants among these ten individuals. The specific gene and variant combinations are detailed at the individual-patient level in Supplementary Table S4.

Two patients who underwent MLPA followed by next-generation sequencing were found to carry large genomic rearrangements: a heterozygous 1.7-kb deletion involving exons 1 and 2 of *BRCA1* and a duplication involving exons 5–11 of *BRCA2*. Detailed variant-level information is provided in Supplementary Table S4.

Genetic diversity indices indicated high gene richness with moderate unevenness: the Shannon diversity index was 2.23, the Simpson diversity index (1−D) was 0.826, and Pielou’s evenness was 0.656. Despite a richness of 30 genes, Hill numbers indicated effective diversities of 9.3 genes at *q* = 1 and 5.8 genes at *q* = 2. The q1/q0 ratio of 0.31 was consistent with a structured long-tail distribution, in which a limited core gene set accounted for most P/LP variants, while numerous low-frequency genes contributed to overall genetic richness.

In the sensitivity analysis restricted to the 333 patients who underwent the common 100-gene NGS panel, richness remained unchanged at 30 categories, whereas diversity and evenness increased. The Shannon index was 2.60, the Simpson index (1−D) was 0.899, and Pielou’s evenness was 0.765. Hill effective diversity increased to 13.5 categories at *q* = 1 and 9.9 at *q* = 2, with a q1/q0 ratio of 0.45 (Table S3a). Thus, uniform testing preserved the breadth of the observed spectrum but revealed a less concentrated distribution of positive findings.

In addition to the broad gene-level heterogeneity, we identified several P/LP that are well described as founder variants in specific populations. These included the Ashkenazi Jewish *BRCA1 c.68_69del (185delAG)* [28] and the Eastern European *BRCA1 c.5266dup (5382insC)* [29,30]. We also observed the Brazilian founder *TP53 p.R337H* [31,32,33], alongside other recurrent variants previously reported in diverse geographic contexts: *BRCA1 c.514del* described in Southern Italy [34]; *PALB2 c.715del* recurrent in the Japanese population [35]; *BRCA1 c.188T>A*, a major founder variant in Japan [36]; *BRCA2 c.7480C>T* reported in Korean cohorts [37]; *BRCA1 c.3770_3771del* described in South Asian populations [38]; *BRCA1 c.798_799del* found in North African/Maghrebi populations [39]; and *BRCA1 c.815_824dup*, a West African founder variant also identified in African American and Latin American cohorts [40,41]. One individual carried the *BRCA2* exon 5–11 duplication, first described in the Israeli Arab Christian population [42] (Figure 3 and Supplementary Table S4).

### 2.3. Histopathological Characteristics, Proliferation, Invasions, and Immune Microenvironment

Invasive ductal carcinoma was the predominant histological subtype (*n* = 408, 81.1%), followed by invasive lobular carcinoma (*n* = 39, 7.8%; Table 1; Supplementary Table S3b). Histological subtype distribution differed significantly across germline genetic groups (*p* < 0.001) (Supplementary Table S3b).

High-grade tumors dominated: 52.7% of 482 evaluable tumors were SBR grade III (Supplementary Tables S5 and S6), and 70.8% of 469 were nuclear grade III (Supplementary Tables S7 and S8). Both grade distributions differed significantly across germline genetic groups (*p* < 0.001 for both; Table 2). Ki-67 also differed across genetic groups (Kruskal–Wallis χ^2^ = 76.9; df = 7; *p* < 0.001), with the highest median values in *BRCA1*-associated tumors (Figure 4). Lymphovascular invasion occurred in 54 of 482 evaluable tumors (11.2%); owing to the limited number of events, invasion outcomes were analyzed primarily descriptively.

TIL distribution differed across germline genetic groups (*p* = 0.007). In gene-specific analyses, *BRCA1* carriers had higher odds of moderate or intense TILs than non-carriers (OR 2.63, 95% CI 1.66–4.16; FDR-adjusted *p* < 0.001; Supplementary Table S9).

Luminal phenotypes predominated (*n* = 314, 62.4%), followed by triple-negative phenotypes, including HER2-low tumors (*n* = 170, 33.8%; Table 1; Supplementary Tables S10 and S11). Molecular phenotype was associated with germline genetic group (*p* < 0.001; Cramér’s V = 0.312; Table 3). *BRCA1* was descriptively strongly enriched in triple-negative tumors, whereas *TP53* and *ATM* were enriched in luminal B/HER2-positive tumors, and *CHEK2*, *BRCA2*, and *PALB2* in luminal/HER2-low phenotypes (Table 3; Figure 4).

### 2.4. Survival Outcomes

During the 60-month follow-up window, 16 deaths and 20 local recurrences were recorded in the overall cohort. Among 500 patients evaluated for local recurrence-free survival (LRFS), 20 local recurrence events (4.0%) occurred within 60 months. No statistically significant difference in LRFS was detected across genetic groups (log-rank χ^2^ = 8.21, df = 8; *p* = 0.413; Figure S2). The PFS analysis included all 503 patients and comprised 50 first events: 46 documented progressions, including local recurrences, and four deaths without prior progression. The estimated 60-month PFS was 87.7% (95% CI, 84.5–91.0%). In the exploratory analysis including all genetic groups, no statistically significant difference was detected (log-rank χ^2^ = 5.3, df = 10; *p* = 0.870). Similarly, the comparison restricted to the six major susceptibility genes shown in Figure S3 did not detect a statistically significant difference (log-rank *p* = 0.88).

In the overall cohort, the estimated 60-month overall survival was 95.9% (95% CI, 93.4–97.5%). Among the 426 patients carrying P/LP variants in the six major susceptibility genes, 12 deaths occurred within 60 months. No statistically significant difference in overall survival was detected across *BRCA1*, *BRCA2*, *TP53*, *PALB2*, *CHEK2*, and *ATM* (log-rank χ^2^ = 3.23, df = 5; *p* = 0.664; Figure S4).

All survival analyses were exploratory. The limited number of events and the small size of several genetic groups substantially reduced statistical power and precision; therefore, the non-significant results should not be interpreted as evidence of equivalent survival outcomes across genotypes.

## 3. Discussion

To the best of our knowledge, this is one of the largest Brazilian studies integrating germline findings with detailed clinicopathological data in hereditary breast cancer. Two principal findings emerged from this single-center referral cohort. First, the observed germline architecture was markedly heterogeneous and followed a structured long-tail distribution, with a limited number of recurrent genes coexisting with numerous low-frequency alterations. Second, exploratory genotype–phenotype analyses identified biologically plausible patterns across the major susceptibility genes. These findings provide descriptive evidence from a clinically ascertained Brazilian cohort and may inform the design of future studies evaluating hereditary cancer testing strategies.

Access to germline genetic testing remains limited and uneven in Brazil, particularly within the public health system, where comprehensive hereditary cancer testing has historically not been routinely available. Similar limitations in access to specialized genetic services have been reported across Latin America [8,12,14,16,17,19]. Within this context, the predominance of self-identified White patients in the present cohort, contrasting with national census data in which Brown (Parda) individuals constitute the largest self-reported race population group [43], may be consistent with differential access or referral to specialized oncogenetic care. However, the present study did not assess referral pathways, socioeconomic characteristics, geographic accessibility, insurance status, healthcare-use patterns, or barriers to testing. The cohort composition therefore cannot, by itself, demonstrate that unequal access caused the observed demographic difference. The center’s referral profile, clinical eligibility criteria, regional population structure, and patterns of healthcare use may also have contributed.

Self-reported racial or ethnic categories, population-level admixture, and individual genomic ancestry are related but non-equivalent concepts. Self-identification is an imperfect proxy for genomic ancestry, which may vary substantially among individuals within the same self-reported group [8,12,15]. Previous studies suggest that genomic ancestry may influence variant frequencies, founder effects, and penetrance in *TP53*, *BRCA1/2*, *CHEK2*, and other DNA-repair genes [4,6,8,11,15,44,45]. Nevertheless, because individual genomic ancestry was not assessed, the present study cannot establish ancestry–variant associations or attribute the observed germline spectrum to specific ancestral contributions. Comparison with national demographic data should therefore be interpreted as contextual and hypothesis-generating rather than as a direct measure of ancestry or a causal analysis of access inequality.

The recent incorporation of germline *BRCA1/2* testing into the Brazilian public health system represents an important policy response to a documented gap in hereditary cancer care [46]. Its implementation, reach, and capacity to reduce existing access barriers remain to be evaluated. Although this policy represents substantial progress, restriction to *BRCA1/2* may leave clinically relevant findings in other susceptibility genes undetected. The implications of broader testing for the public health system require prospective evaluation of diagnostic yield, clinical actionability, infrastructure requirements, and cost-effectiveness.

Among individuals who underwent germline testing at the institution, 5.0% met the study eligibility criteria. This percentage should not be interpreted as the prevalence of hereditary breast cancer or P/LP variants among all tested individuals because inclusion required breast cancer and a qualifying germline finding. The median age at breast cancer diagnosis was 42 years and varied across genetic groups, with *BRCA1* carriers being diagnosed a median of six years earlier than patients in the rare-gene group. This pattern is consistent with previous evidence that *BRCA1*-associated breast cancer frequently presents at a younger age than disease associated with several other susceptibility genes [1,21,24].

Family history remained an important driver of referral, although nearly one-third of patients reported no family history of cancer. This observation is consistent with evidence that pedigree-based criteria alone may fail to identify some P/LP variant carriers because of small family size, adoption, incomplete documentation, reduced penetrance, sex-limited phenotypes, or paternal inheritance [6,8,15]. It supports continued evaluation of testing criteria that integrate personal cancer characteristics with family history rather than relying exclusively on pedigree information.

The occurrence of multiple primary tumors highlights the relevance of surveillance beyond the initial breast cancer diagnosis. The tumor spectrum observed among *TP53* carriers, including carriers of the Brazilian founder variant *TP53* c.1010G>A (p.R337H), was consistent with the recognized clinical spectrum of Li–Fraumeni syndrome [32,33,47,48,49]. Findings among *BRCA1/2* carriers were also compatible with established recommendations concerning contralateral breast surveillance, consideration of risk-reducing procedures, and screening for associated malignancies [1,21,23,24]. The contribution of *ATM*, *CHEK2*, and *PALB2* further illustrates that hereditary breast cancer assessment extends beyond *BRCA1/2*, although evidence remains insufficient to support gene-specific surveillance recommendations for several rare genes [2,5,6,24].

The germline landscape was dominated by *BRCA1*, *BRCA2*, and *TP53*, followed by *ATM*, *PALB2*, and *CHEK2*, while numerous low-frequency genes contributed to overall richness. This distribution is consistent with previous Brazilian and Latin American multigene studies and supports a model in which a limited set of recurrent genes coexists with a long tail of rare alterations [4,14,18,20,25]. The founder-variant spectrum provides additional historical context. Variants previously reported in European, Ashkenazi Jewish, Asian, African, Middle Eastern, and South Asian populations were observed alongside the Brazilian founder variant *TP53* c.1010G>A (p.R337H; rs121912651) [11,12,28,29,30,31,34]. Their occurrence is compatible with the migration and gene-flow processes that contributed to the demographic formation of Brazil. However, because neither individual genomic ancestry nor variant-associated haplotypes was assessed, the study cannot reconstruct the routes through which these alleles entered the cohort or establish ancestry–variant relationships at the individual level. Within this Brazilian referral cohort, the findings illustrate the limitations of interpreting hereditary cancer variation solely through evidence derived from predominantly European-ancestry populations [14,18].

The diversity metrics provided a quantitative description of the observed germline distribution. In the complete cohort, approximately 30 genetic categories corresponded to effective diversities of 9.3 at q = 1 and 5.8 at q = 2, indicating that most observations were concentrated in a smaller set of common genes while numerous rare categories formed a long tail. However, these estimates were derived from P/LP-positive patients who underwent heterogeneous testing pathways and should not be interpreted as population-level measures of hereditary breast cancer architecture. In particular, sequential *BRCA1/2* testing could increase the relative representation of these genes while reducing the opportunity to identify additional findings in less frequently assessed genes [2,8].

The sensitivity analysis restricted to 333 patients tested with a common 100-gene panel retained the same observed richness but showed greater evenness and effective diversity. The Shannon index increased from 2.23 to 2.60, the Simpson index from 0.826 to 0.899, and Pielou’s evenness from 0.656 to 0.765; Hill numbers increased from 9.3 to 13.5 at q = 1 and from 5.8 to 9.9 at q = 2. This comparison indicates that testing architecture influenced the relative concentration of the observed distribution, particularly the predominance of *BRCA1/2*, while the broad long-tail spectrum remained evident under uniform multigene testing. The results support the value of comprehensive testing for genetic characterization, but they do not establish its incremental clinical yield or cost-effectiveness.

The observed distribution suggests a candidate eight-gene testing framework comprising *BRCA1*, *BRCA2*, *TP53*, *PALB2*, *CHEK2*, *ATM*, *RAD51C*, and *RAD51D*. These genes combined recurrent detection in the cohort with established clinical relevance. Nevertheless, this framework is hypothesis-generating and should not be interpreted as a definitive panel recommendation. Panel selection must also consider penetrance, clinical actionability, analytical performance, incremental diagnostic yield, testing coverage, counselling requirements, and health-economic consequences. Prospective studies using uniform gene coverage are therefore required before this candidate framework can inform clinical implementation or health policy [2,8].

The unadjusted genotype–phenotype analyses identified structured patterns in tumor characteristics. *BRCA1*-associated tumors were enriched for triple-negative phenotype, higher histological and nuclear grade, elevated Ki-67, and increased tumor-infiltrating lymphocytes. These findings are biologically compatible with homologous recombination deficiency and previously recognized immunogenic features of *BRCA1*-associated disease [1,23,50,51]. In contrast, *TP53*- and *ATM*-associated tumors showed greater representation of luminal B or HER2-positive phenotypes, whereas *PALB2-*, *BRCA2-*, and *CHEK2*-associated tumors more frequently showed luminal or HER2-low phenotypes [3,6,7,10,21,22,52,53]. The apparent enrichment of MMR variants in luminal A/HER2-low tumors should be interpreted particularly cautiously because of the small number of cases.

These genotype–phenotype findings should not be interpreted as independent genetic effects. The analyses were exploratory and unadjusted, and residual confounding by age at diagnosis and other clinicopathological characteristics cannot be excluded. Tumor phenotype, grade, Ki-67, and TILs are also biologically and statistically interrelated, while several genetic and pathological categories contained small numbers of observations. The results therefore describe patterns observed in this cohort and require confirmation in larger, uniformly tested datasets using prospectively specified multivariable models. Although the patterns are consistent with relationships between germline genotype and tumor biology described in previous studies [3,4,44,45,54,55], their independent prognostic or therapeutic implications cannot be established from the present analysis.

The survival analyses were likewise exploratory. Within 60 months, 50 first PFS events and 16 deaths occurred in the complete cohort, while comparisons among the six major susceptibility genes included only 42 PFS events and 12 deaths. No statistically significant differences in PFS or OS were detected among these genes. These findings should not be interpreted as evidence of equivalent survival across genotypes because the low event counts and small size of several groups produced limited statistical power and imprecise estimates. Longer follow-up and larger cohorts are required to evaluate genotype-specific survival outcomes.

Overall, the findings may inform future research on regionally adapted hereditary cancer testing, but their applicability beyond this cohort requires cautious evaluation. Brazil has substantial regional demographic and healthcare-system heterogeneity, and Latin American populations differ markedly in demographic history, genomic backgrounds, referral practices, access to genetic testing, and healthcare organization. Multicenter studies using uniform genetic testing, broader geographic representation, direct genomic ancestry assessment, and evaluation of socioeconomic and health-system factors are needed before specific testing strategies can be recommended at national or regional levels [2,11,14,19].

## 4. Limitations

This study was conducted at a single tertiary cancer referral center, and the cohort should not be considered representative of Brazil or Latin America. Although unequal access to germline testing is a recognized challenge in Brazil, referral pathways, socioeconomic characteristics, geographic accessibility, insurance status, and barriers to testing were not directly assessed. Therefore, differences between the cohort’s self-reported racial or ethnic composition and national census data cannot be causally attributed to unequal access. Individual genomic ancestry was also not assessed; consequently, self-reported racial or ethnic categories cannot support ancestry–variant associations or definitive ancestry-specific interpretations of founder variants.

Genetic testing strategies and gene-level coverage were heterogeneous and varied according to clinical indication, test availability, and insurance authorization. Patients tested initially for *BRCA1/2* generally underwent broader testing only when the initial findings were negative and hereditary cancer suspicion remained. This differential testing opportunity may have resulted in the underdetection of variants outside *BRCA1/2*, including additional multilocus findings, and may have influenced the observed gene frequencies and diversity estimates. Although the sensitivity analysis of 333 patients tested with a common 100-gene panel reduced this concern, it did not eliminate referral or ascertainment bias. MLPA was also not performed systematically, and additional large genomic rearrangements may have remained undetected. The findings should therefore be interpreted as descriptive of this clinically ascertained P/LP-positive cohort rather than as population-level prevalence estimates.

The ambispective design included a substantial retrospective component, and selected clinicopathological variables were incomplete. Rare genes were grouped into predefined biologically related categories to reduce sparse-data bias, which may have obscured individual gene effects. Genotype–phenotype analyses were exploratory and unadjusted; residual confounding by age at diagnosis and other clinicopathological characteristics cannot be excluded. The limited sample sizes of several genetic and outcome categories and the correlation among tumor phenotype, grade, Ki-67, and TILs restricted the reliability of post hoc multivariable modelling. Similarly, survival analyses were limited by the low number of events and small subgroup sizes. Non-significant log-rank results should therefore not be interpreted as evidence of equivalent survival across genotypes.

Tumor mutational burden could not be evaluated because tumor sequencing data were unavailable. The study also did not assess the incremental diagnostic yield, clinical actionability, cost-effectiveness, or budget impact of alternative testing strategies. Accordingly, the proposed eight-gene framework should be regarded as hypothesis-generating rather than as a definitive clinical panel recommendation and requires prospective validation using uniform gene coverage and formal health-economic evaluation.

## 5. Methods

### 5.1. Study Design and Setting

We conducted a single-center ambispective cohort study including patients with breast cancer evaluated at the Oncogenetics Outpatient Clinic of A.C.Camargo Cancer Center (Fundação Antonio Prudente), a tertiary cancer referral center in Brazil, between January 2019 and July 2025. Data from patients evaluated between January 2019 and 21 April 2025, were collected retrospectively from institutional medical records, whereas patients evaluated from 22 April 2025 through July 2025 constituted the prospective component. Both components followed the same eligibility criteria and were integrated into a single database and analyzed jointly.

This study was conducted in accordance with the Declaration of Helsinki and approved by the Research Ethics Committee of A.C.Camargo Cancer Center (CAAE 85806925.8.0000.5432) on 22 April 2025. The requirement for informed consent was waived by the Research Ethics Committee because the study used anonymized data and involved no study-related interventions.

### 5.2. Study Population

Eligible participants were adults (≥18 years) with histologically confirmed invasive or microinvasive breast cancer and a P/LP germline variant (P/LP variant) in a cancer predisposition gene, classified according to the criteria of the American College of Medical Genetics and Genomics and the Association for Molecular Pathology (ACMG/AMP). For variants with conflicting classifications across cancer phenotypes, the classification supported by evidence specific to breast cancer predisposition was used.

Individuals with carcinoma in situ only, variants of uncertain significance, asymptomatic carriers, or insufficient clinical or pathological data were excluded. Detailed eligibility criteria are provided in the Supplementary Methods S1 and S2. The study flow is shown in Supplementary Figure S1.

### 5.3. Data Collection and Variables

Clinical, pathological, and molecular data were extracted from electronic medical records. Variables included age at diagnosis, sex, self-reported race or ethnicity, family history of cancer, synchronous or metachronous tumors, tumor stage (AJCC TNM, 8th edition) [56], histological subtype, Scarff–Bloom–Richardson (SBR) grade, Nottingham nuclear grade, Ki-67 proliferation index, lymphovascular, blood vessel and perineural invasion, tumor-infiltrating lymphocytes (TILs) and molecular phenotype. Detailed information is available in the Supplementary Materials.

For selected analyses, participants self-identifying as Black (“Preta”) or Brown (“Parda”) according to the Brazilian Institute of Geography and Statistics (IBGE) classification were combined into a single “Afro-descendant” group because of the limited number of Black participants.

### 5.4. Genetic Testing and Gene Grouping

Germline genetic testing followed heterogeneous, real-world clinical pathways. Of the 503 patients, 333 (66.2%) underwent multigene NGS testing using a common 100-gene panel with copy number variation (CNV) analysis. The complete panel composition is provided in Supplementary Table S13. Among the remaining patients, 151 underwent complete sequencing of *BRCA1/2*, 17 underwent targeted testing for a specific *BRCA1* or *BRCA2* variant, and two underwent MLPA followed by NGS.

Testing strategies varied according to clinical indication, test availability, and insurance coverage. In patients initially tested for *BRCA1/2*, broader multigene panel testing was generally considered when the initial results were negative and clinical suspicion of hereditary cancer remained. When a clinically relevant P/LP variant was identified through a restricted testing strategy, additional testing was not routinely performed. Consequently, gene-level coverage was not uniform across the complete cohort, and patients did not have an equivalent opportunity for P/LP variants to be detected in every gene.

Genes with adequate sample sizes for individual analysis (*BRCA1*, *BRCA2*, *TP53*, *PALB2*, *CHEK2*, and *ATM*) were analyzed separately. Less frequent breast cancer predisposition genes were grouped into biologically and clinically defined categories: the RAD-associated group (*RAD51C*, *RAD51D*, and *XRCC2*), mismatch repair genes (*MLH1*, *MSH2*, *MSH6*, and *PMS2*), the MRN complex (*RAD50*, *MRE11*, and *NBN*), and other rare genes. Multilocus genotypes, including multilocus inherited neoplasia allele syndrome (MINAS), were handled according to predefined analytical rules detailed in the Supplementary Methods S3–S10.

### 5.5. Outcome-Oriented Analytical Strategy

The analytical strategy was predefined according to the biological characteristics and frequency of each outcome. Genes with sufficient sample size were analyzed individually for outcomes with clear ordinal structure and prognostic relevance, including SBR grade, nuclear grade, and Ki-67.

Outcomes generating sparse contingency tables, including histological subtypes and less frequent pathological variables, were analyzed using predefined biologically related gene groups (RAD family, MMR genes, MRN complex, and other rare genes) to improve statistical stability. Analyses of tumor invasion were restricted to lymphovascular invasion, whereas blood vessel and perineural invasion were analyzed descriptively because of the limited number of events. This strategy was adopted as *a priori* to ensure robust and biologically meaningful statistical inference.

The analytical unit for clinicopathological and tumor-characteristic analyses was the patient, and only one index breast tumor was included per patient. For patients with metachronous breast cancers, the first diagnosed tumor was selected. For patients with synchronous or multifocal tumors, the largest invasive tumor was selected; when tumor size did not clearly determine the index lesion, the tumor with the higher histological grade or more aggressive molecular phenotype was used. Thus, multiple tumors from the same patient did not contribute independently to the same analysis.

### 5.6. Genetic Diversity Analysis

To characterize the observed distribution of P/LP-positive findings, diversity metrics adapted from ecology were applied to the complete cohort. A sensitivity analysis was additionally performed in the 333 patients who underwent the same 100-gene NGS panel with CNV analysis to assess the influence of differential gene-level ascertainment. Both analyses used the same predefined genetic-category coding to ensure direct comparability. Diversity was quantified using richness (q = 0), the Shannon diversity index (H′), Simpson diversity (1−D), Pielou’s evenness, and Hill numbers. Hill diversity at q = 1 was calculated as exp(H′), whereas diversity at q = 2 was calculated as the inverse Simpson concentration. The q1/q0 ratio was used to describe the proportion of observed richness represented by effective diversity. Additional methodological details and analytical-category definitions are provided in the Supplementary Methods S11 and S12.

### 5.7. Statistical Analysis

Categorical variables are presented as frequencies and percentages; continuous variables as means (SD) or medians (IQR), as appropriate. Group comparisons for continuous variables used the Kruskal–Wallis test with Dunn’s post-hoc test (adjusted for multiple comparisons when applicable), and median differences were estimated using the Hodges–Lehmann estimator. Associations between categorical variables were assessed using Pearson’s χ^2^ test (with Cramér’s V for effect size) or Fisher’s exact test with Monte Carlo simulation when expected frequencies were low. All tests were two-sided, with *p* < 0.05 considered statistically significant. Analyses were performed using R version 4.3.1.

### 5.8. Survival Analysis

Overall survival (OS), progression-free survival (PFS), and local recurrence-free survival (LRFS) were estimated using the Kaplan–Meier method, with 95% confidence intervals. Exploratory comparisons across genetic groups were performed using the log-rank test. OS was defined as the interval from breast cancer diagnosis to death from any cause. PFS was defined as the interval from diagnosis to the first documented disease progression, including local recurrence, or death from any cause, whichever occurred first. LRFS was defined as the interval from definitive treatment to the first documented local recurrence.

For each endpoint, follow-up was calculated from the corresponding starting point to the first event or the last documented clinical assessment. Patients without an event, including those lost to follow-up, were censored at the date of their last documented assessment. Follow-up was administratively censored at 60 months. Given the limited number of events and the small size of several genetic groups, survival comparisons were considered exploratory, and no multivariable survival models were fitted.

## 6. Conclusions

This study identified a heterogeneous germline spectrum and clinically coherent genotype–phenotype associations within a Brazilian referral cohort of patients with hereditary breast cancer. The concentration of clinically relevant variants in a core group of susceptibility genes, together with a long tail of rare alterations that persisted in the uniformly tested subset, provides a descriptive basis for future research on broader, regionally adapted multigene testing.

Because testing coverage was heterogeneous and individual genomic ancestry was not assessed, the findings reflect this clinically ascertained cohort rather than population-level prevalence or ancestry-specific estimates. The proposed panel composition represents a candidate framework for prospective validation using uniform genetic testing, broader geographic representation, assessment of incremental diagnostic yield and clinical actionability, and formal health-economic evaluation before national or regional implementation.

## Figures and Tables

**Figure 1:**
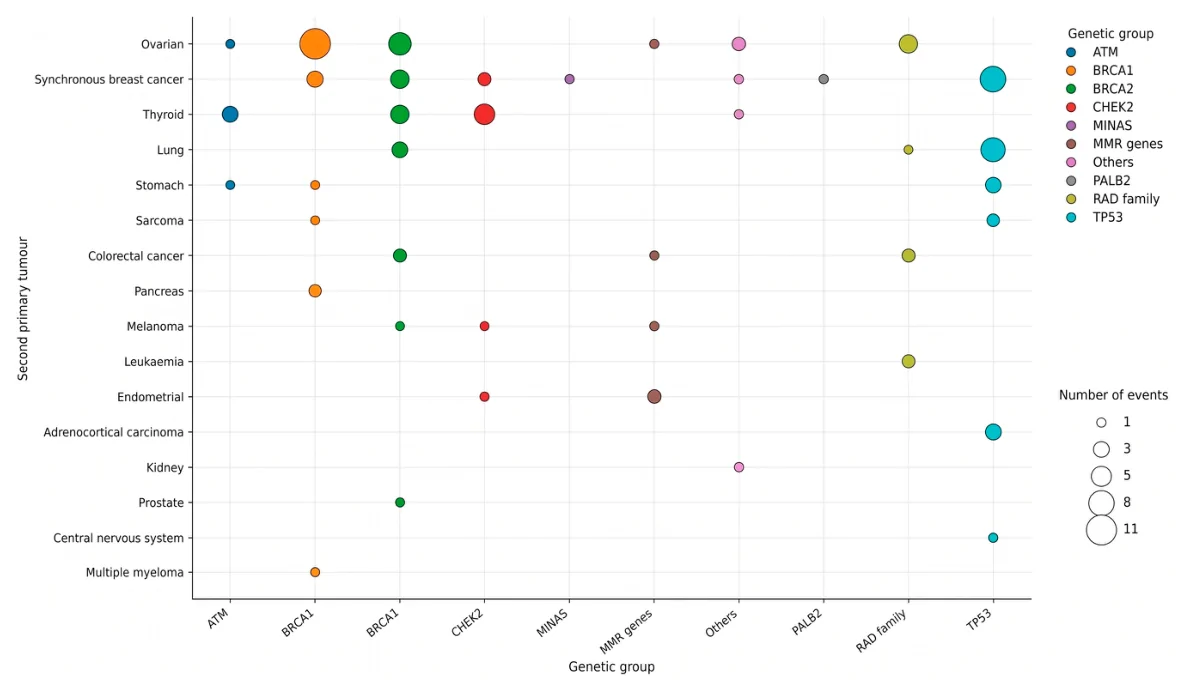
**Distribution of synchronous tumors across germline genetic groups.** Bubble size represents the number of synchronous tumor events observed for each tumor type within each germline genetic group. Synchronous breast cancer refers to a second primary breast cancer. Genetic groups are shown only when at least one synchronous tumor was observed. MMR = mismatch repair; MINAS = multilocus inherited neoplasia allele syndrome.

**Figure 2:**
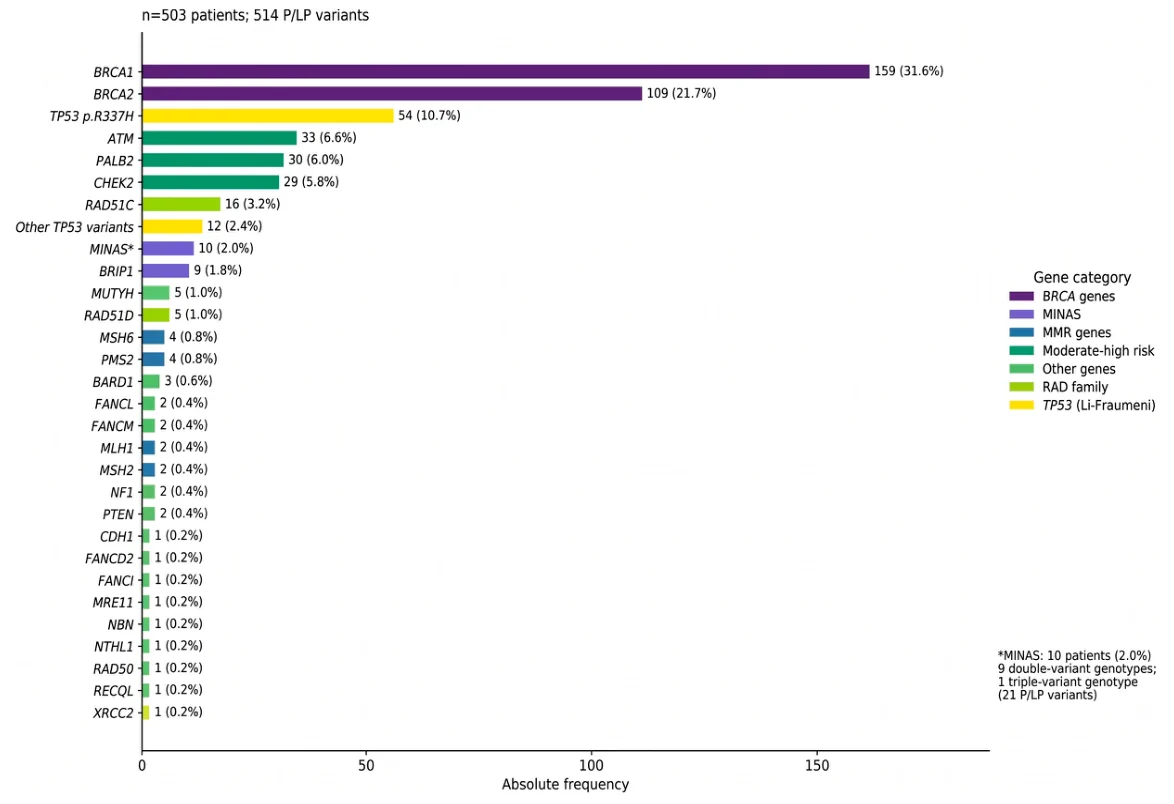
**Distribution of germline P/LP variants among patients with hereditary breast cancer.** Bars represent the number and proportion of patients carrying a P/LP variant in each gene or genetic category. Percentages were calculated using the total cohort (*n* = 503) as the denominator. * MINAS comprised ten patients with multilocus inherited neoplasia allele syndrome, including nine with two P/LP variants and one with three P/LP variants, corresponding to 21 variants overall. In total, 514 P/LP variants were identified among 503 patients. MMR = mismatch repair; MINAS = multilocus inherited neoplasia allele syndrome; P/LP = pathogenic or likely pathogenic.

**Figure 3:**
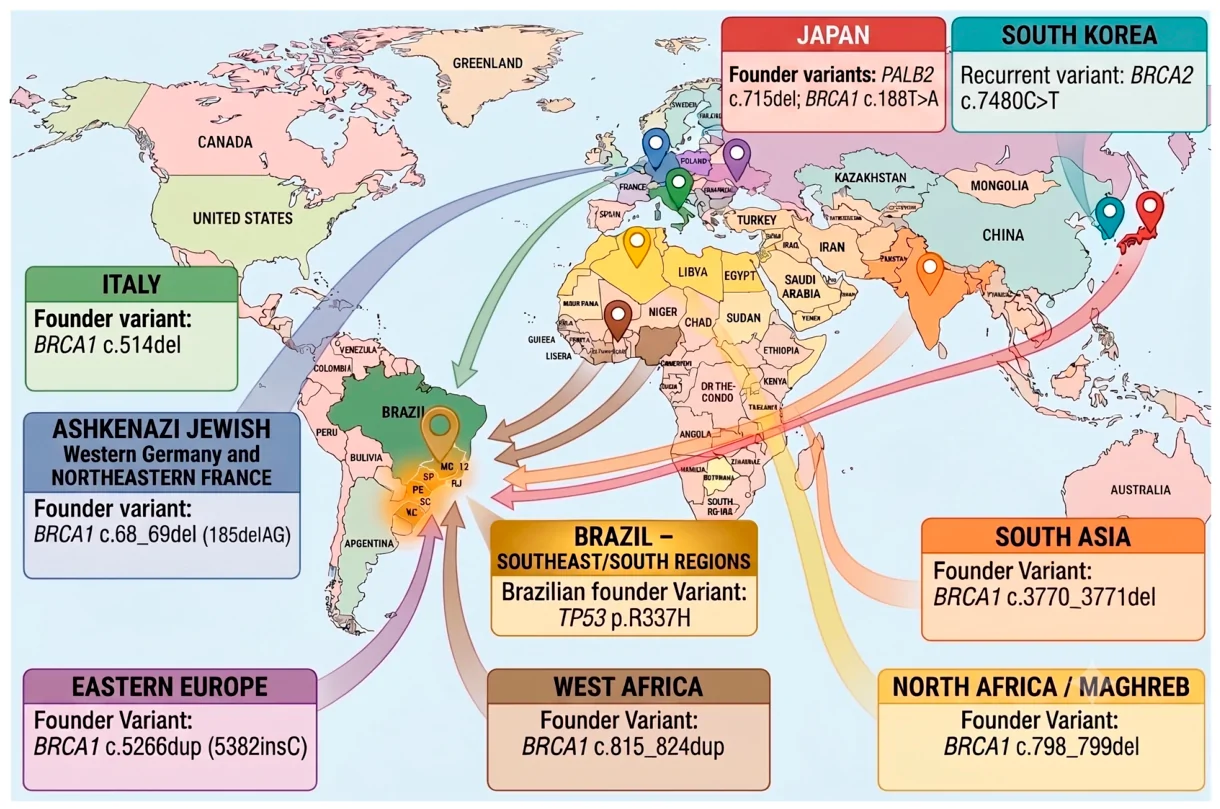
**Previously reported geographic origins and historical dispersal contexts of recurrent founder variants identified in the Brazilian cohort.** Geographic assignments and migration routes are based on published population-genetic and historical evidence cited in the manuscript. Arrows schematically represent historically documented or previously proposed population movements and do not indicate individual genomic ancestry, migration histories, or ancestry reconstruction among participants in the present cohort.

**Figure 4:**
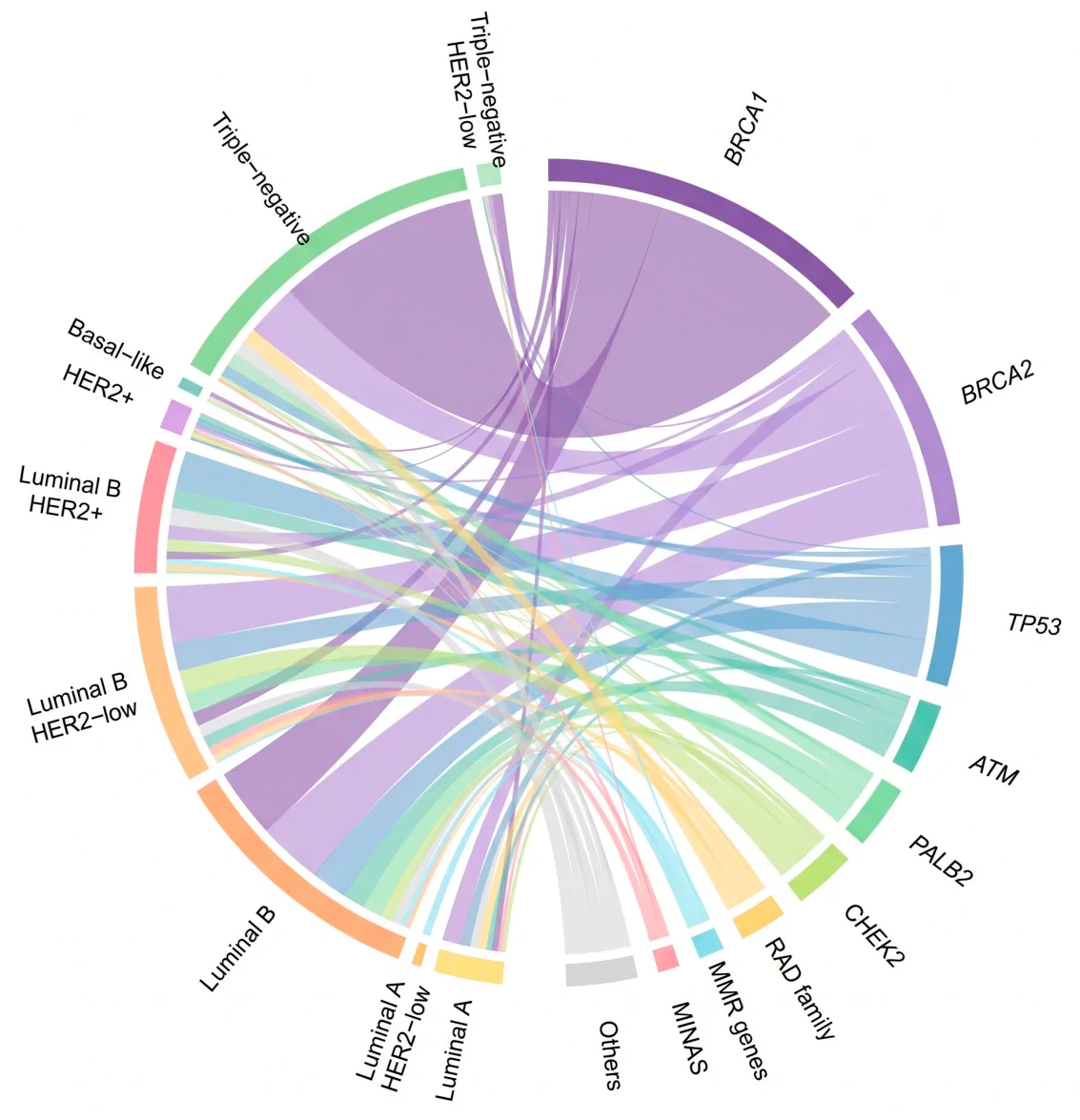
**Distribution of molecular phenotypes across germline gene groups.** Chord width represents the number of patients within each germline genetic group–tumor phenotype combination. Genetic groups are displayed on the right and tumor molecular phenotypes on the left. Link colors correspond to the respective germline genetic groups. MMR = mismatch repair; MINAS = multilocus inherited neoplasia allele syndrome; HER2 = human epidermal growth factor receptor 2.

**Table 1:** Clinical and epidemiological characteristics of patients with hereditary breast cancer (*n* = 503).

Characteristics	*n* (%) or Median (IQR)
**Sex**	
Female	501 (99.6)
Male	2 (0.4)
**Age at diagnosis, years**	**42 (36–49)**
<30 years	25 (5.0)
30–39 years	173 (34.4)
40–49 years	181 (36.0)
50–59 years	85 (16.9)
≥60 years	39 (7.8)
**Family history of cancer**	
Yes	354 (70.4)
No	149 (29.6)
**Tumor category at diagnosis**	
T1–T2	399 (79.8)
T3–T4	101 (20.2)
**Nodal status**	
N0	307 (61.6)
N1–N2	178 (35.7)
N3	13 (2.6)
**Clinical stage**	
Stage I–II	397 (79.4)
Stage III	94 (18.8)
Stage IV	9 (1.8)
**Histological subtype**	
Invasive ductal carcinoma	408 (81.1)
Invasive lobular carcinoma	39 (7.8)
Other subtypes	56 (11.1)
**Molecular phenotype**	
Triple-negative (including HER2-low)	170 (33.8)
Luminal A (including HER2-low)	36 (7.2)
Luminal B (all categories)	278 (55.3)
HER2-positive non-luminal	14 (2.7)
Basal-like	5 (1.0)

Data are presented as *n* (%) unless otherwise indicated. Age at diagnosis is presented as median (IQR). Percentages may not total 100% because of rounding. Tumor category and clinical stage were classified according to the AJCC TNM staging system (8th edition). Percentages were calculated among participants with available data: tumor category, *n* = 500; nodal status, *n* = 498; and clinical stage, *n* = 500.

**Table 2:** Tumor grade, proliferation, lymphovascular invasion, and immune microenvironment.

Variable	Overall, *n* (%) or Median (IQR)	Global *p* Value
**SBR grade (** ***n*** ** = 482)**		**<0.001**
Grade I	32 (6.6)	
Grade II	196 (40.7)	
Grade III	254 (52.7)	
**Nuclear grade (** ***n*** ** = 469)**		**<0.001**
Grade I	15 (3.2)	
Grade II	122 (26.0)	
Grade III	332 (70.8)	
**Ki-67, % (** ***n*** ** = 462)**	**50 (30–70)**	**<0.001**
**Lymphovascular invasion (** ***n*** ** = 482)**	54 (11.2)	—
**Tumor-infiltrating lymphocytes (** ***n*** ** = 392)**		**0.007**
Low	254 (64.8)	
Moderate or intense (High)	138 (35.2)	

Data are presented as *n* (%) or median (IQR). Global associations across germline genetic groups were assessed using Fisher’s exact test with Monte Carlo simulation for categorical variables and the Kruskal–Wallis test for continuous variables. Ki-67 analyses were restricted to genetic groups with at least ten informative cases. Lymphovascular invasion is presented descriptively because of the limited number of events. SBR = Scarff–Bloom–Richardson; IQR = interquartile range.

**Table 3:** Association between germline genetic groups and tumor molecular phenotypes.

Germline Genetic Group	Enriched Phenotypes (z)	Depleted Phenotypes (z)
***BRCA1***	Triple-negative (+10.59)	Luminal HER2-low (−5.53); Luminal B/HER2-positive (−4.55); Luminal A (−2.80)
***TP53***, including p.R337H	Luminal B/HER2-positive (+4.36)	Triple-negative (−4.25)
***ATM***	Luminal B/HER2-positive (+3.25)	Triple-negative (−4.06)
***CHEK2***	Luminal HER2-low (+3.27)	Triple-negative (−2.97)
***BRCA2***	Luminal HER2-low (+2.47)	Luminal B/HER2-positive (−2.12)
***PALB2***	Luminal HER2-low (+2.16)	—
***RAD *** **family**	—	Luminal B (−2.79)
**MMR genes**	Luminal A/HER2-low (+12.84)	Triple-negative (−2.39)

**Global association:** Fisher’s exact test with Monte Carlo simulation, *p* < 0.001; Cramér’s V = 0.312. Only gene group–phenotype combinations with absolute standardized residuals |z| ≥ 2 are shown. Positive values indicate enrichment and negative values indicate depletion. The *RAD* family comprises *RAD51C*, *RAD51D*, and *XRCC2*. Mismatch repair (MMR) genes comprise *MLH1*, *MSH2*, *MSH6*, and *PMS2*. HER2 = human epidermal growth factor receptor 2. Standardized residuals were used descriptively to identify the cells contributing to a significant global association and should not be interpreted as independently tested or adjusted associations.

## Data Availability

Deidentified participant-level germline variant data are provided in Supplementary Table S4. Additional deidentified data underlying the study findings may be made available upon reasonable request to the corresponding author, subject to approval by the institutional Research Ethics Committee and applicable data protection regulations. The study protocol and statistical analysis code may also be made available upon reasonable request. The complete clinical dataset will not be publicly deposited because of the sensitive and potentially identifiable nature of combined clinical and germline genetic information.

## Supplementary Materials

The following supporting information can be downloaded at: https://www.mdpi.com/article/10.3390/ijms27219686/s1.
