# Supplementary material for: Hereditary Breast Cancer in a Brazilian Referral Cohort: Germline Diversity and Genotype–Phenotype Associations
Source: Int J Mol Sci. 2026 Oct 29;27(21):9686. doi: 10.3390/ijms27219686 (PMC13763213; doi:10.3390/ijms27219686)
Supplement: Supplementary file 1 [file ijms-27-09686-s001.zip › ijms-4518274-supplementary.pdf]

Supplementary Material

Hereditary Breast Cancer in a Brazilian Referral Cohort: Germline Diversity and Genotype–Phenotype Associations

André Luiz Cicilini; Patrick Vinicius Cerqueira Batista; Sandrine M Caputo; Viviane Primo Basilio de Souza; Solange Moraes Sanchez; Fabiana Baroni Makdissi; Marina de Brot ; Marina de Assis da Escossia Fernandes; Beatriz Nelli Barbatto; Leticia Coelho de Mattos; Nathalia Machado Soldi; Flavia Cavalcanti Ballint; Débora Guilherme de Albuquerque e Rodrigues de Sousa; Eduarda Mirella da Silva Montiel; Lurick Rodrigues Soares; Thamilyn Yoshizaki Saruwatari; Sahlua Miguel Volc; Solange Maria Torchia Carvalho Castro; Giovana Tardin Torrezan; Dirce Maria Carraro; Alexandre Andre Balliero; Vladimir Claudio Cordeiro de Lima; José Claudio Casali-da-Rocha e Elizabeth Santana dos Santos.

Correspondence to:

José Claudio Casali-da-Rocha, MD, PhD

casali.rocha@accamargo.org.br

Elizabeth Santana dos Santos, MD, PhD

elizabeth.santanadossantos@gmail.com

Supplementary Methods

S1. Eligibility criteria

Patients were eligible if they met all of the following criteria:

- age ≥18 years at diagnosis;
- histologically confirmed invasive or microinvasive breast cancer;
- presence of a pathogenic or likely pathogenic (P/LP) germline variant in a cancer predisposition gene, classified according to the criteria of the American College of Medical Genetics and Genomics and the Association for Molecular Pathology (ACMG/AMP). For variants with conflicting classifications across cancer phenotypes, the classification supported by evidence specific to breast cancer predisposition was used.

Exclusion criteria were:

- asymptomatic P/LP variant carriers without a breast cancer diagnosis;
- variants of uncertain significance (VUS) without sufficient evidence supporting pathogenicity in the context of breast cancer predisposition;
- non-invasive breast lesions, including ductal carcinoma in situ and atypical hyperplasia;
- insufficient, inconsistent, or unreliable clinical or pathological data.

These criteria were applied to define a clinically homogeneous cohort suitable for genotype–phenotype and outcome analyses.

S2. Study population and data flow

During the study period, 10,098 individuals underwent germline genetic testing at the Oncogenetics Outpatient Clinic of A.C. Camargo Cancer Center. Following systematic application of the eligibility criteria, individuals without invasive or microinvasive breast cancer, asymptomatic carriers, those harboring only VUS, and those with insufficient clinical or pathological data were excluded. The final analytic cohort comprised 503 patients with invasive or microinvasive breast cancer and at least one P/LP germline variant. The complete study selection process is shown in Supplementary Figure S1.

Figure S1. Study flow diagram

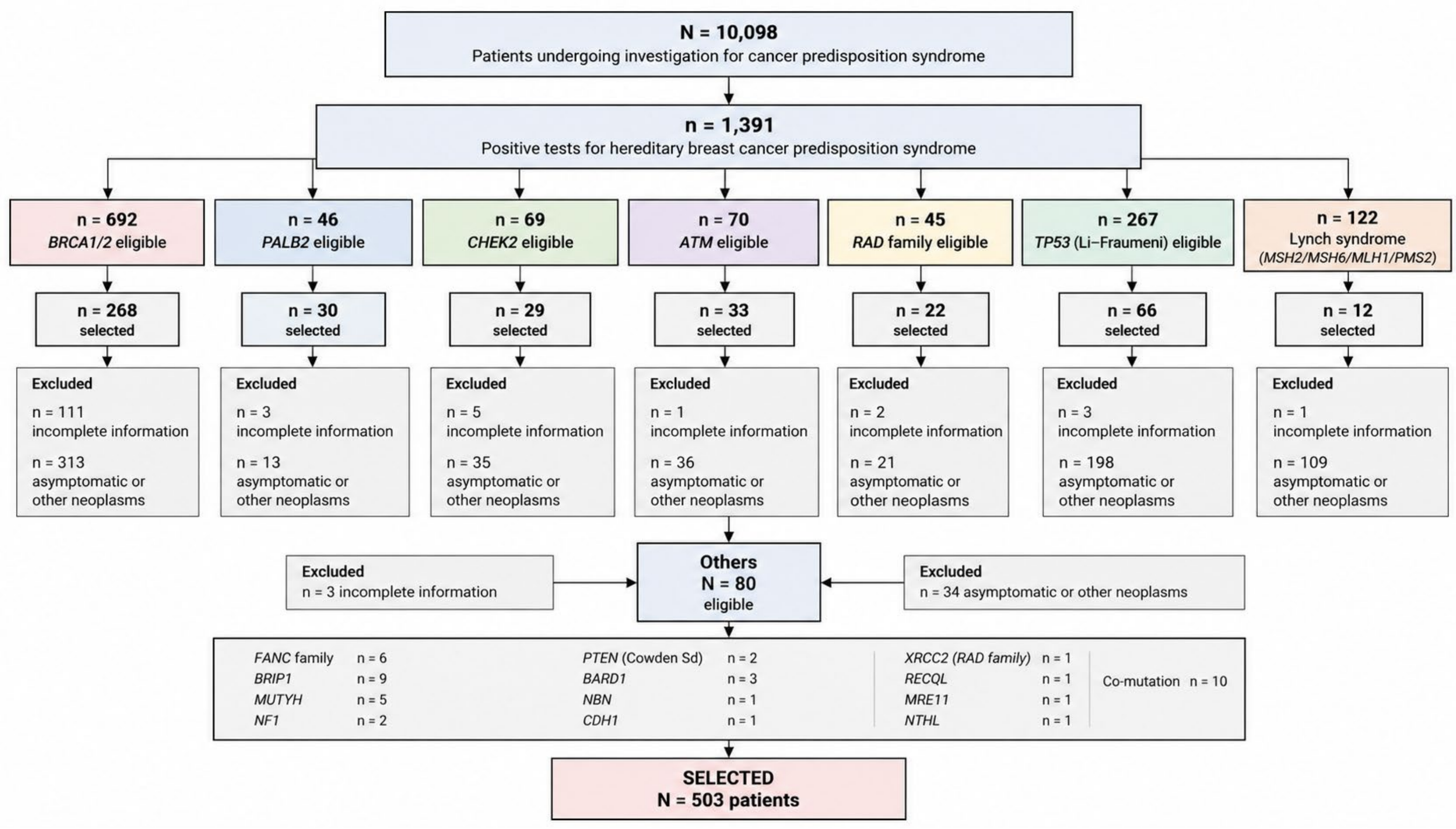

S3. Genetic testing procedures

Germline genetic testing was performed using either sequential BRCA1/2 testing followed, when indicated, by multigene panel sequencing, or multigene next-generation sequencing (NGS) panels with copy number variation (CNV) analysis. In two patients, BRCA1/2 testing consisted of MLPA followed by NGS. Testing strategies and panel composition varied according to clinical indication, test availability, and insurance coverage; consequently, not all genes were assessed in all patients.

Genetic testing followed real-world clinical pathways. In many patients, BRCA1/2 testing was performed first, with broader multigene panel testing considered when BRCA1/2 results were negative and the clinical suspicion of hereditary cancer remained. Access to broader testing also depended on insurance authorization or the ability to self-fund testing. When a clinically relevant P/LP variant was identified using a more restricted testing strategy, additional testing was not routinely performed.

S4. Variant classification

Germline variants were classified according to the criteria of the American College of Medical Genetics and Genomics and the Association for Molecular Pathology (ACMG/AMP). Variant interpretation incorporated evidence from population and disease-specific databases, published literature, computational prediction tools, functional evidence, and segregation data, when available. Only pathogenic or likely pathogenic (P/LP) variants were included. Variants of uncertain significance were excluded. For variants with conflicting classifications across cancer phenotypes, the classification supported by evidence specific to breast cancer predisposition was used.

S5. Definition and grouping of germline genes

Genes with sufficient sample size for stable statistical inference—*BRCA1, BRCA2, TP53, PALB2,CHEK2,* and *ATM*—were analyzed individually. Less frequent breast cancer predisposition genes were grouped into predefined biologically and clinically related categories to reduce sparse-data bias and improve interpretability. The RAD family comprised *RAD51C, RAD51D,* and *XRCC2*; mismatch repair (MMR) genes comprised *MLH1, MSH2, MSH6,* and *PMS2*; and the MRN complex comprised *RAD50, MRE11,* and *NBN.* Remaining low-frequency genes were included in the "Others" category. The Brazilian founder variant *TP53* c.1010G>A (p.R337H) was evaluated separately in selected descriptive analyses.

S6. Handling of multilocus pathogenic variants

Multilocus inherited neoplasia allele syndrome (MINAS) was defined as the presence of pathogenic or likely pathogenic germline variants in two or more cancer predisposition genes in the same individual. Patients with MINAS were analyzed as a distinct genetic category. Specific gene combinations were additionally reported in descriptive analyses. The cohort included ten patients with MINAS: nine carried two P/LP variants and one carried three P/LP variants, corresponding to 21 P/LP variants overall.

S7. Clinical and pathological variables

Clinical stage was defined according to the American Joint Committee on Cancer (AJCC) TNM classification, 8th edition. Histological subtype was assigned according to institutional pathology reports. Histological grade was assessed using the Scarff–Bloom–Richardson (SBR) system and nuclear grade according to Nottingham criteria. Tumor invasion was recorded as lymphovascular, blood vessel, or perineural invasion; these categories were not mutually exclusive.

S8. Molecular phenotypes

Tumor molecular phenotypes were classified using immunohistochemical surrogate definitions based on estrogen receptor (ER), progesterone receptor (PR), HER2 expression, the Ki-67 proliferation index, and basal marker expression. Hormone receptor positivity was defined as nuclear staining in ≥1% of tumor cells. Low proliferative activity was defined as Ki-67 <20%, whereas Ki-67 ≥20% was considered high. HER2-zero was defined as an immunohistochemical score of 0; HER2-low as a score of 1+ or 2+ with negative in situ hybridization; and HER2-positive as a score of 3+ or 2+ with HER2 amplification by in situ hybridization.

Luminal A HER2-zero was defined as ER-positive and/or PR-positive, HER2-zero, and Ki-67 <20%; Luminal A HER2-low as ER-positive and/or PR-positive, HER2-low, and Ki-67 <20%; Luminal B HER2-zero as ER-positive and/or PR-positive, HER2-zero, and Ki-67 ≥20%; Luminal B HER2-low as ER-positive and/or PR-positive, HER2-low, and Ki-67 ≥20%; and Luminal B HER2-positive as ER-positive and/or PR-positive and HER2-positive, regardless of the Ki-67 index. HER2-positive non-luminal tumors were ER-negative, PR-negative, and HER2-positive.

Among ER-negative and PR-negative tumors, the core basal-like phenotype was confirmed by pathological assessment based on positive immunohistochemical expression of cytokeratin 5/6 (CK5/6) and/or epidermal growth factor receptor (EGFR). These tumors were classified exclusively as basal-like. ER-negative and PR-negative tumors without CK5/6 or EGFR expression were classified as triple-negative HER2-zero or triple-negative HER2-low according to their HER2 immunohistochemical status.

Accordingly, tumors were assigned to one of nine mutually exclusive molecular phenotype categories: Luminal A HER2-zero, Luminal A HER2-low, Luminal B HER2-zero, Luminal B HER2-low, Luminal B HER2-positive, HER2-positive non-luminal, triple-negative HER2-zero, triple-negative HER2-low, or basal-like. Phenotype distributions are presented in Supplementary Tables S10 and S11.

S9. Tumor-infiltrating lymphocytes

Tumor-infiltrating lymphocytes (TILs) were evaluated on hematoxylin and eosin-stained sections according to international consensus recommendations. TILs were categorized as discrete (<10%), moderate (10–40%), or intense (>40%). For inferential analyses, TILs were dichotomized as low (discrete) versus high (moderate or intense).

S10. Outcome-oriented analytical strategy

The analytical strategy was predefined according to the biological characteristics, distribution, and frequency of each outcome. Genes with adequate sample sizes were analyzed individually. Outcome-specific methods were selected according to whether variables were categorical, ordinal, or continuous, including SBR grade, nuclear grade, and Ki-67.

Outcomes generating sparse contingency tables, including histological subtypes and less frequent pathological variables, were analyzed using predefined biologically related gene groups to improve statistical stability and interpretability. Analyses of tumor invasion were restricted to lymphovascular invasion, whereas blood vessel and perineural invasion were presented descriptively because of the limited number of events. The genotype–phenotype analyses were exploratory and unadjusted and were not intended to estimate independent genetic effects.

S11. Genetic diversity analysis

Genetic diversity was evaluated using predefined patient-level genetic categories. Each patient contributed one observation to the analysis. The Brazilian founder variant TP53 p.R337H was retained as a separate category from other TP53 variants, and patients with multilocus inherited neoplasia allele syndrome (MINAS) were assigned to a distinct multilocus category. Accordingly, these metrics describe the diversity and relative distribution of the positive genetic categories observed in the cohort rather than variant-level diversity or population-level gene prevalence.

- For each genetic category i, relative abundance was calculated as: $p_i = n_i/N$
- where n_i represents the number of patients assigned to category i, and N represents the total number of patients included in the analysis.
- Genetic-category richness corresponded to the number of observed categories (S) and was equivalent to the Hill number of order zero: D(0) = S
- The Shannon diversity index was calculated as: $H' = -\Sigma\, p_i \ln(p_i)$
- where the summation includes all observed genetic categories and ln denotes the natural logarithm.
- Simpson concentration was calculated as: $\lambda = \Sigma\, p_i^2$
- Simpson diversity was subsequently expressed as: $1 - \lambda = 1 - \Sigma\, p_i^2$
- This index represents the probability that two randomly selected patients belonged to different genetic categories. Pielou's evenness was calculated as: $J' = H'/\ln(S)$

- Hill numbers were used to express diversity as the effective number of equally abundant genetic categories. Hill diversity of order one was calculated as the exponential of Shannon entropy:
- $D(1) = \exp(H')$
- Hill diversity of order two was calculated as the inverse of Simpson concentration: $D(2) = 1/\Sigma\ p_i^2$
- The ratio between the effective number of common categories and the observed genetic-category richness was calculated as: $D(1)/D(0)$

This ratio describes the proportion of observed genetic-category richness contributing meaningfully to effective diversity, with lower values indicating a more uneven distribution dominated by a limited number of common categories.

The primary analysis included all 503 patients and therefore reflected the heterogeneous testing pathways encountered in clinical practice. To evaluate the influence of differential gene-level ascertainment, a sensitivity analysis was restricted to the 333 patients who underwent the same 100-gene NGS panel with CNV analysis. Identical genetic-category definitions and diversity calculations were applied to both analyses to ensure direct comparability.

S12. Statistical analysis

Statistical analyses were performed using R version 4.3.1. Categorical variables are presented as frequencies and percentages, and continuous variables as means with standard deviations or medians with interquartile ranges, as appropriate. Continuous variables were compared across genetic groups using the Kruskal–Wallis test, followed by Dunn's post-hoc test with adjustment for multiple comparisons when indicated. Median differences were estimated using the Hodges–Lehmann estimator.

Associations between categorical variables were assessed using Pearson's χ^2 test, with Cramér's V used to estimate effect size, or Fisher's exact test with Monte Carlo simulation when expected frequencies were low. Adjusted standardized residuals were examined to identify enriched or depleted gene–phenotype combinations. For analyses involving multiple gene-specific comparisons, p values were adjusted using the false discovery rate method. All tests were two-sided, and $p < 0.05$ was considered statistically significant.

Supplementary Results

Table S1 - Age at diagnosis by genetic group

Germline genetic group	N	Mean, years	Median, years	Q1	Q3
BRCA1	159	41.7	41.0	35.0	46.0
BRCA2	109	45.2	43.0	38.0	51.0
TP53	66	43.1	40.0	35.0	50.0
ATM	33	43.3	41.0	37.0	48.0
PALB2	30	41.7	41.0	36.0	45.0
CHEK2	29	43.5	42.0	36.0	52.0
RAD family	22	48.9	49.0	39.0	59.0
MMR genes	12	48.0	42.0	39.0	58.0
MINAS	10	41.2	42.0	38.0	45.0
Others	33	47.0	42.5	42.0	55.5

Data are presented as means, medians, and interquartile ranges (Q1–Q3). Age at breast cancer diagnosis was available for all 503 patients. RAD family includes *RAD51C, RAD51D,* and *XRCC2*. MMR=mismatch repair; MINAS=multilocus inherited neoplasia allele syndrome.

Table S2. Spectrum of synchronous tumors according to germline genetic group

Germline genetic group	Synchronous tumors observed, n events
ATM	Thyroid (n = 3); stomach (n = 1); ovarian (n = 1)
BRCA1	Ovarian (n = 11); synchronous breast cancer (n = 3); pancreatic (n = 2); stomach (n = 1); multiple myeloma (n = 1); sarcoma (n = 1)
BRCA2	Ovarian (n = 6); synchronous breast cancer (n = 4); thyroid (n = 4); lung (n = 3); colorectal (n = 2); melanoma (n = 1); prostate (n = 1)
CHEK2	Thyroid (n = 5); synchronous breast cancer (n = 2); endometrial (n = 1); melanoma (n = 1)
MINAS	Synchronous breast cancer (n = 1)
MMR genes	Endometrial (n = 2); colorectal (n = 1); ovarian (n = 1); melanoma (n = 1)
Others	Ovarian (n = 2); synchronous breast cancer (n = 1); kidney (n = 1); thyroid (n = 1)
PALB2	Synchronous breast cancer (n = 1)
RAD family	Ovarian (n = 4); leukemia (n = 2); colorectal (n = 2); lung (n = 1)
TP53	Synchronous breast cancer (n = 8); lung (n = 7); adrenocortical carcinoma (n = 3); stomach (n = 3); sarcoma (n = 2); central nervous system tumor (n = 1)

Data represents the number of synchronous tumor events rather than the number of affected patients. Individual patients could contribute more than one synchronous tumor event. RAD family includes *RAD51C, RAD51D,* and *XRCC2*. MMR=mismatch repair; MINAS=multilocus inherited neoplasia allele syndrome.

Table S3. Distribution of patients according to germline gene or genetic group

Gene or genetic group	N	Proportion (%)
BRCA1	159	31.6
BRCA2	109	21.7
**TP53 (total)**	**66**	**13.1**
└ *TP53* p.R337H	54	10.7
└ Other *TP53* variants	12	2.4
ATM	33	6.6
PALB2	30	6.0
CHEK2	29	5.8
**RAD family (total)**	**22**	**4.4**
└ *RAD51C*	16	3.2
└ *RAD51D*	5	1.0
└ *XRCC2*	1	0.2
**MMR genes (total)**	**12**	**2.4**
└ *MSH6*	4	0.8
└ *PMS2*	4	0.8
└ *MLH1*	2	0.4
└ *MSH2*	2	0.4
MINAS	10	2.0
BRIP1	9	1.8
MUTYH	5	1.0
BARD1	3	0.6
FANCL	2	0.4
FANCM	2	0.4
NF1	2	0.4
PTEN	2	0.4

Table S3. continued

Gene or genetic group	N	Proportion (%)
**MRN complex genes (total)**	**3**	**0.6**
└ *RAD50*	1	0.2
└ *MRE11*	1	0.2
└ *NBN*	1	0.2
CDH1	1	0.2
FANCD2	1	0.2
FANCI	1	0.2
NTHL1	1	0.2
RECQL	1	0.2
**Total**	**503**	**100.0**

Data represents the number and proportion of patients in each mutually exclusive genetic category; percentages were calculated using the total cohort (n = 503) as the denominator. Indented gene-level categories are components of the corresponding aggregate groups and are not additional observations. The RAD family comprises *RAD51C, RAD51D,* and *XRCC2*; MMR genes comprise *MLH1, MSH2, MSH6,* and *PMS2*; and MRN complex genes comprise *RAD50, MRE11,* and *NBN*. MINAS denotes multilocus inherited neoplasia allele syndrome and comprised ten patients: nine with two P/LP variants and one with three P/LP variants. Overall, 514 P/LP variants were identified among 503 patients. P/LP=pathogenic or likely pathogenic; MMR=mismatch repair; MINAS=multilocus inherited neoplasia allele syndrome.

Table S3a- Comparison of genetic-category diversity metrics between the complete clinically ascertained cohort and the uniformly tested subset.

**Diversity metric**	**Complete cohort, N = 503**	**Uniform 100-gene panel, N = 333**
Richness, q = 0	30.0	30.0
Shannon index, H′	2.23	2.60
Simpson diversity, 1−D	0.826	0.899
Pielou's evenness	0.656	0.765
Hill number, q = 1	9.3	13.5
Hill number, q = 2	5.8	9.9
q1/q0 ratio	0.31	0.45

The sensitivity analysis included 333 patients who underwent the same 100-gene NGS panel with CNV analysis. Both analyses used identical predefined genetic-category definitions.

Supplementary Table S3b Distribution of histological subtype according to germline genetic group

Germline genetic group	Invasive ductal carcinoma	Invasive lobular carcinoma	Metaplastic/medullary carcinoma	Other histological subtypes	Total
BRCA1	139 (87.4)	1 (0.6)	13 (8.2)	6 (3.8)	159
BRCA2	87 (79.8)	13 (11.9)	1 (0.9)	8 (7.3)	109
TP53	51 (77.3)	7 (10.6)	2 (3.0)	6 (9.1)	66
PALB2	23 (76.7)	3 (10.0)	0 (0.0)	4 (13.3)	30
CHEK2	25 (86.2)	2 (6.9)	0 (0.0)	2 (6.9)	29
Others	83 (75.5)	13 (11.8)	1 (0.9)	13 (11.8)	110
Overall	408 (81.1)	39 (7.8)	17 (3.4)	39 (7.8)	503

Data are presented as n (row %). Histological subtypes were grouped for inferential analysis because of sparse cell counts. The “Other histological subtypes” category included mixed, mucinous, micropapillary, apocrine, papillary, pleomorphic lobular, salivary gland–type, acinar, myxoid, and pleomorphic carcinomas. Histological subtype distribution differed significantly across germline genetic groups (Fisher’s exact test with Monte Carlo simulation, $p < 0.001$). Histological subtype information was available for all 503 patients. The Others genetic group included *ATM*, RAD family, MMR genes, MINAS, and other low-frequency genes.

Table S4. Distribution of patients according to germline gene or genetic group

ID	Gee	Chr:Pos (GRCh38)	Variant	Type	dbsnp	gnomad	Clinvar/ ACMG	Comment
SIGILO1	*BRCA1*	17:43124028-43124031	NM_007294.4(BRCA1):c.68_69del (p.Glu23fs)	frameshift	rs80357914	0.0002054	Pathogenic	Ashkenazi Jewish founder variant[1]
SIGILO2	*BRCA1*	17: 43092809-43092812	NM_007294.4(BRCA1):c.2719_2722delGAAG (p.E907Kfs*92)	frameshift	rs80357731	ND	Pathogenic	
SIGILO3	*BRCA1*	17: 43106457	NM_007294.4(BRCA1):c.211A>G (p.Arg71Gly)	missense	rs80357382	0.000004004	Pathogenic	
SIGILO4	*BRCA1*	17: 43092439-43092440	NM_007294.4(BRCA1):c.3091dup (p.Ile1031fs)	frameshift	rs1555588399	ND	Pathogenic	
SIGILO5	*BRCA1*	17: 43063903	NM_007294.4(BRCA1):c.5123C>A (p.Ala1708Glu)	missense	rs28897696	0.00040	Pathogenic	
SIGILO6	*BRCA1*	17: 43067606	NM_007294.4(BRCA1):c.5074+2T>C	splice donor	rs80358089	ND	Pathogenic	
SIGILO7	*BRCA1*	17: 43093844	NM_007294.4(BRCA1):c.1687C>T (p.Gln563Ter)	nonsense	rs80356898	0.00002795	Pathogenic	
SIGILO8	*BRCA1*	17: 43093844	NM_007294.4(BRCA1):c.1687C>T (p.Q563*)	nonsense	rs80356898	0.00002795	Pathogenic	
SIGILO9	*BRCA1*	17:43124030-43124031	NM_007294.4(BRCA1):c.66dup (p.Glu23fs)	frameshift	rs80357783	0.00001	Pathogenic	Ashkenazi Jewish founder variant[1]
SIGILO10	*BRCA1*	17: 43093570	NM_007294.4(BRCA1):c.1961del (p.Lys654fs)	frameshift	rs80357522	0.00001063	Pathogenic	
SIGILO11	*BRCA1*	17: 43057062-43057063	NM_007294.4(BRCA1):c.5266dupC (p.Gln1756Profs)	frameshift	rs80357906	0.0001803	Pathogenic	Eastern European founder variant (5382insC)[2,3]
SIGILO12	*BRCA1*	17: 43106457	NM_007294.4(BRCA1):c.211A>G (p.Arg71Gly)	missense	rs80357382	0.000004004	Pathogenic	
SIGILO13	*BRCA1*	17: 43092197-43092200	NM_007294.4(BRCA1):c.3331_3334del (p.Gln1111fs)	frameshift	rs80357701	ND	Pathogenic	
SIGILO14	*BRCA1*	17: 43092274	NM_007294.4(BRCA1):c.3257T>G (p.Leu1086Ter)	nonsense	rs80357006	ND	Pathogenic	
SIGILO15	*BRCA1*	17: 43091012	NM_007294.4(BRCA1):c.4117G>T (p.Glu1373Ter)	nonsense	rs80357259	ND	Pathogenic	
SIGILO16	*BRCA1*	17: 43104156	NM_007294.4(BRCA1):c.407del (p.Arg136fs)	frameshift	rs886040200	ND	Pathogenic	
SIGILO17	*BRCA1*	17: 43106478	NM_007294.4(BRCA1):c.190T>C (p.Cys64Arg)	missense	rs80357064	ND	Pathogenic	
SIGILO18	*BRCA1*	17: 43106457	NM_007294.4(BRCA1):c.211A>G (p.Arg71Gly)	missense	rs80357382	0.000004004	Pathogenic	
SIGILO19	*BRCA1*	17: 43093316	NM_007294.4(BRCA1):c.2215A>T (p.Lys739Ter)	nonsense	rs56329598	ND	Pathogenic	
SIGILO20	*BRCA1*	17: 43106480	NM_007294.4(BRCA1):c.188T>A (p.Leu63Ter)	nonsense	rs80357086	ND	Pathogenic	Japanese founder variant (≈30% of BRCA1 carriers in Japan)[4]
SIGILO21	*BRCA1*	17: 43092197-43092200	NM_007294.4(BRCA1):c.3331_3334del (p.Gln1111fs)	frameshift	rs80357701	ND	Pathogenic	
SIGILO22	*BRCA1*	17: 43092197-43092200	NM_007294.4(BRCA1):c.3331_3334del (p.Gln1111fs)	frameshift	rs80357701	ND	Pathogenic	
SIGILO23	*BRCA1*	17: 43092197-43092200	NM_007294.4(BRCA1):c.3331_3334del (p.Gln1111fs)	frameshift	rs80357701	ND	Pathogenic	
SIGILO24	*BRCA1*	17: 43057062-43057063	NM_007294.4(BRCA1):c.5266dupC (p.Gln1756Profs)	frameshift	rs80357906	0.0001803	Pathogenic	
SIGILO25	*BRCA1*	17: 43093844	NM_007294.4(BRCA1):c.1687C>T (p.Gln563Ter)	nonsense	rs80356898	0.00002795	Pathogenic	
SIGILO26	*BRCA1*	17: 43047665	NM_007294.4(BRCA1):c.5445G>A (p.Trp1815Ter)	nonsense	rs397509284	0.000003976	Pathogenic	
SIGILO27	*BRCA1*	17: 43082474	NM_007294.4(BRCA1):c.4287C>A (p.Tyr1429Ter)	nonsense	rs397509160	ND	Pathogenic	
SIGILO28	*BRCA1*	17: 43099851-43099852	NM_007294.4(BRCA1):c.470_471del (p.Leu156_Ser157insTer)	nonsense	rs80357887	ND	Pathogenic	
SIGILO29	*BRCA1*	17: 43067606	NM_007294.4(BRCA1):c.5074+2T>C	splice donor	rs80358089	ND	Pathogenic	
SIGILO30	*BRCA1*	17: 43057062-43057063	NM_007294.4(BRCA1):c.5266dup (p.Gln1756fs)	frameshift	rs80357906	0.0001803	Pathogenic	
SIGILO31	*BRCA1*	17: 43094732-43094733	NM_007294.4(BRCA1):c.798_799del (p.Ser267fs)	frameshift	rs80357724	ND	Pathogenic	Described in North African/Maghrebi populations[5]
SIGILO32	*BRCA1*	17: 43094706-43094707	NM_007294.4(BRCA1):c.815_824dup (p.Thr276fs)	frameshift	rs387906563	ND	Pathogenic	West African founder variant; identified in Senegalese, African American, and Latin American cohorts[6,7]

Table S4. continued

ID	Gee	Chr:Pos (GRCh38)	Variant	Type	dbsnp	gnomad	Clinvar/ ACMG	Comment
SIGILO33	BRCA1	17: 43104120	NM_007294.4(BRCA1):c.441+2T>A	splice donor	rs397509173	ND	Pathogenic / Likely Pathogenic	
SIGILO34	BRCA1	17: 43076492	NM_007294.4(BRCA1):c.4480G>T (p.Glu1494Ter)	nonsense	rs80357148	ND	Pathogenic	
SIGILO35	BRCA1	17: 43067606	NM_007294.4(BRCA1):c.5074+2T>A	splice donor	rs80358089	ND	Pathogenic	
SIGILO36	BRCA1	17: 43063369	NM_007294.4(BRCA1):c.5157del (p.Thr1720fs)	frameshift	rs2549384286	ND	Pathogenic	
SIGILO37	BRCA1	17: 43057078	NM_007294.4(BRCA1):c.5251C>T (p.Arg1751Ter)	nonsense	rs80357123	ND	Pathogenic	
SIGILO38	BRCA1	17: 43057062-43057063	NM_007294.4(BRCA1):c.5266dup (p.Gln1756fs)	frameshift	rs80357906	0.0001803	Pathogenic	
SIGILO39	BRCA1	17: 43057062-43057063	NM_007294.4(BRCA1):c.5266dup (p.Gln1756fs)	frameshift	rs80357906	0.0001803	Pathogenic	
SIGILO40	*BRCA1*	17: 43057062-43057063	NM_007294.4(BRCA1):c.5266dup (p.Gln1756fs)	frameshift	rs80357906	0.0001803	Pathogenic	
SIGILO41	*BRCA1*	17: 43051091	NM_007294.4(BRCA1):c.5304del (p.Tyr1769fs)	frameshift	rs80357959	ND	Pathogenic	
SIGILO42	*BRCA1*	17: 43057062-43057063	NM_007294.4(BRCA1):c.5266dup (p.Gln1756fs)	frameshift	rs80357906	0.0001803	Pathogenic	
SIGILO43	*BRCA1*	17: 43057062-43057063	NM_007294.4(BRCA1):c.5266dup (p.Gln1756fs)	frameshift	rs80357906	0.0001803	Pathogenic	
SIGILO44	*BRCA1*	17: 43099773	NM_007294.4(BRCA1):c.547+2T>A	splice donor	rs80358047	ND	Pathogenic	
SIGILO45	*BRCA1*	17:43124028-43124031	NM_007294.4(BRCA1):c.68_69del (p.Glu23fs)	frameshift	rs80357914	0.0002054	Pathogenic	
SIGILO46	*BRCA1*	17: 43067606	NM_007294.4(BRCA1):c.5074+2T>C	splice donor	rs80358089	ND	Pathogenic	
SIGILO47	*BRCA1*	17: 43067606	NM_007294.4(BRCA1):c.5074+2T>C	splice donor	rs80358089	ND	Pathogenic	
SIGILO48	*BRCA1*	17: 43067606	NM_007294.4(BRCA1):c.5074+2T>C	splice donor	rs80358089	ND	Pathogenic	
SIGILO49	*BRCA1*	17: 43124030-43124031	NM_007294.4(BRCA1):c.66dup (p.Glu23fs)	frameshift	rs80357783	0.00001	Pathogenic	
SIGILO50	*BRCA1*	17: 43093844	NM_007294.4(BRCA1):c.1687C>T (p.Gln563Ter)	nonsense	rs80356898	0.00002795	Pathogenic	
SIGILO51	*BRCA1*	17:43124028-43124031	NM_007294.4(BRCA1):c.68_69del (p.Glu23fs)	frameshift	rs80357914	0.0002054	Pathogenic	
SIGILO52	*BRCA1*	17: 43092731	NM_007294.4(BRCA1):c.2800C>T (p.Gln934Ter)	nonsense	rs80357223	ND	Pathogenic	
SIGILO53	*BRCA1*	17: 43057052-43076614	NM_007294.3(BRCA1):c.4358-?_5277+?del	deletion (exonic)	----	ND	Pathogenic	
SIGILO54	*BRCA1*	17: 43067606	NM_007294.4(BRCA1):c.5074+2T>C	splice donor	rs80358089	ND	Pathogenic	
SIGILO55	*BRCA1*	17: 43106457	NM_007294.4(BRCA1):c.211A>G (p.Arg71Gly)	missense	rs80357382	0.000004004	Pathogenic	
SIGILO56	*BRCA1*	17: 43057052-43076614	NM_007294.3(BRCA1):c.4358-?_5277+?del	deletion (exonic)	----	ND	Pathogenic	
SIGILO57	*BRCA1*	17: 43092197-43092200	NM_007294.4(BRCA1):c.3331_3334del (p.Gln1111fs)	frameshift	rs80357701	ND	Pathogenic	
SIGILO58	*BRCA1*	17: 43067606	NM_007294.4(BRCA1):c.5074+2T>C	splice donor	rs80358089	ND	Pathogenic	
SIGILO59	*BRCA1*	17: 43106480	NM_007294.4(BRCA1):c.188T>A (p.Leu63Ter)	nonsense	rs80357086	ND	Pathogenic	
SIGILO60	*BRCA1*	17: 43067663-43067665	NM_007294.4(BRCA1):c.5014CAC[1] (p.His1673del)	inframe deletion	rs80358343	ND	Pathogenic/ Likely-Pathogenic	
SIGILO61	*BRCA1*	17: 43067663-43067665	NM_007294.4(BRCA1):c.5014CAC[1] (p.His1673del)	inframe deletion	rs80358343	ND	Pathogenic/ Likely-Pathogenic	

Table S4. continued

ID	Gee	Chr:Pos (GRCh38)	Variant	Type	dbsnp	gnomad	Clinvar/ ACMG	Comment
SIGILO62	*BRCA1*	17: 43106480	NM_007294.4(BRCA1):c.188T>A (p.Leu63Ter)	nonsense	rs80357086	ND	Pathogenic	
SIGILO63	*BRCA1*	17: 43045766	NM_007294.4(BRCA1):c.5504G>A (p.Arg1835Gln)	missense	rs80356962	ND	Pathogenic	
SIGILO64	*BRCA1*	17: 43057062-43057063	NM_007294.4(BRCA1):c.5266dup (p.Gln1756fs)	frameshift	rs80357906	0.0001803	Pathogenic	
SIGILO65	*BRCA1*	17: 43057062-43057063	NM_007294.4(BRCA1):c.5266dup (p.Gln1756fs)	frameshift	rs80357906	0.0001803	Pathogenic	
SIGILO66	*BRCA1*	17: 43093844	NM_007294.4(BRCA1):c.1687C>T (p.Gln563Ter)	nonsense	rs80356898	0.00002795	Pathogenic	
SIGILO67	*BRCA1*	17: 43091760-43091761	NM_007294.4(BRCA1):c.3770_3771del (p.Glu1257fs)	frameshift	rs80357579	0.00000796 1	Pathogenic	Described in South Asian populations (Pakistan)[8]
SIGILO68	*BRCA1*	17: 43067631	NM_007294.4(BRCA1):c.5051del (p.Thr1684fs)	frameshift	rs1135401881	ND	Pathogenic	
SIGILO69	*BRCA1*	17: 43092197-43092200	NM_007294.4(BRCA1):c.3331_3334del (p.Gln1111fs)	frameshift	rs80357701	ND	Pathogenic	
SIGILO70	*BRCA1*	17: 43092197-43092200	NM_007294.4(BRCA1):c.3331_3334del (p.Gln1111fs)	frameshift	rs80357701	ND	Pathogenic	
SIGILO71	*BRCA1*	17: 43045758	NM_007294.4(BRCA1):c.5512del (p.Val1838fs)	frameshift	rs80357839	ND	Pathogenic	
SIGILO72	*BRCA1*	17: 43045784-43045785	NM_007294.4(BRCA1):c.5485dup (p.Glu1829fs)	frameshift	rs768401297	ND	Pathogenic	
SIGILO73	*BRCA1*	17: 43057062-43057063	NM_007294.4(BRCA1):c.5266dupC (p.Gln1756Profs)	frameshift	rs80357906	0.0001803	Pathogenic	
SIGILO74	*BRCA1*	17: 43057062-43057063	NM_007294.4(BRCA1):c.5266dupC (p.Gln1756Profs)	frameshift	rs80357906	0.0001803	Pathogenic	
SIGILO75	*BRCA1*	17: 43057062-43057063	NM_007294.4(BRCA1):c.5266dup (p.Gln1756fs)	frameshift	rs80357906	0.0001803	Pathogenic	
SIGILO76	*BRCA1*	17: 43057062-43057063	NM_007294.4(BRCA1):c.5266dup (p.Gln1756fs)	frameshift	rs80357906	0.0001803	Pathogenic	
SIGILO77	*BRCA1*	17: 43057062-43057063	NM_007294.4(BRCA1):c.5266dup (p.Gln1756fs)	frameshift	rs80357906	0.0001803	Pathogenic	
SIGILO78	*BRCA1*	17: 43106457	NM_007294.4(BRCA1):c.211A>G (p.Arg71Gly)	missense	rs80357382	0.00000400 4	Pathogenic	
SIGILO79	*BRCA1*	17: 43106457	NM_007294.4(BRCA1):c.211A>G (p.Arg71Gly)	missense	rs80357382	0.00000400 4	Pathogenic	
SIGILO80	*BRCA1*	17: 43093844	NM_007294.4(BRCA1):c.1687C>T (p.Gln563Ter)	nonsense	rs80356898	0.00002795	Pathogenic	
SIGILO81	*BRCA1*	17: 43093844	NM_007294.4(BRCA1):c.1687C>T (p.Gln563Ter)	nonsense	rs80356898	0.00002795	Pathogenic	
SIGILO82	*BRCA1*	17: 43106487	NM_007294.4(BRCA1):c.181T>G (p.Cys61Gly)	missense	rs28897672	0.0000172	Pathogenic	
SIGILO83	*BRCA1*	17: 43057062-43057063	NM_007294.4(BRCA1):c.5266dup (p.Gln1756fs)	frameshift	rs80357906	0.0001803	Pathogenic	
SIGILO84	*BRCA1*	17: 43057062-43057063	NM_007294.4(BRCA1):c.5266dupC (p.Gln1756Profs)	frameshift	rs80357906	0.0001803	Pathogenic	
SIGILO85	*BRCA1*	17: 43094732-43094733	NM_007294.4(BRCA1):c.798_799del (p.Ser267fs)	frameshift	rs80357724	ND	Pathogenic	
SIGILO86	*BRCA1*	17: 43067663-43067665	NM_007294.4(BRCA1):c.5014CAC[1] (p.His1673del)	inframe deletion	rs80358343	ND	Pathogenic/ Likely-Pathogenic	
SIGILO87	*BRCA1*	17: 43067606	NM_007294.4(BRCA1):c.5074+2T>C	splice donor	rs80358089	ND	Pathogenic	
SIGILO88	*BRCA1*	17: 43057062-43057063	NM_007294.4(BRCA1):c.5266dupC (p.Gln1756Profs)	frameshift	rs80357906	0.0001803	Pathogenic	
SIGILO89	*BRCA1*	17: 43067606	NM_007294.4(BRCA1):c.5074+2T>C	splice donor	rs80358089	ND	Pathogenic	
SIGILO90	*BRCA1*	17: 43106457	NM_007294.4(BRCA1):c.211A>G (p.Arg71Gly)	missense	rs80357382	0.00000400 4	Pathogenic	

Table S4. continued

ID	Gee	Chr:Pos (GRCh38)	Variant	Type	dbsnp	gnomad	Clinvar/ ACMG	Comment
SIGILO91	*BRCA1*	17: 43057062-43057063	NM_007294.4(BRCA1):c.5266dupC (p.Gln1756Profs)	frameshift	rs80357906	0.0001803	Pathogenic	
SIGILO92	*BRCA1*	17: 43057062-43057063	NM_007294.4(BRCA1):c.5266dupC (p.Gln1756Profs)	frameshift	rs80357906	0.0001803	Pathogenic	
SIGILO93	*BRCA1*	17: 43099851-43099852	NM_007294.4(BRCA1):c.470_471del (p.Leu156_Ser157insTer)	nonsense	rs80357887	ND	Pathogenic	
SIGILO94	*BRCA1*	17: 43124032	NM_007294.4(BRCA1):c.65T>C (p.Leu22Ser)	missense	rs80357438	ND	Pathogenic	
SIGILO95	*BRCA1*	17: 43067606	NM_007294.4(BRCA1):c.5074+2T>C	splice donor	rs80358089	ND	Pathogenic	
SIGILO96	*BRCA1*	17: 43067606	NM_007294.4(BRCA1):c.5074+2T>C	splice donor	rs80358089	ND	Pathogenic	
SIGILO97	*BRCA1*	17: 43057062-43057063	NM_007294.4(BRCA1):c.5266dupC (p.Gln1756Profs)	frameshift	rs80357906	0.0001803	Pathogenic	
SIGILO98	*BRCA1*	17: 43057062-43057063	NM_007294.4(BRCA1):c.5266dupC (p.Gln1756Profs)	frameshift	rs80357906	0.0001803	Pathogenic	
SIGILO99	*BRCA1*	17: 43057062-43057063	NM_007294.4(BRCA1):c.5266dupC (p.Gln1756Profs)	frameshift	rs80357906	0.0001803	Pathogenic	
SIGILO100	*BRCA1*	17: 43092197-43092200	NM_007294.4(BRCA1):c.3331_3334del (p.Gln1111fs)	frameshift	rs80357701	ND	Pathogenic	
SIGILO101	*BRCA1*	17: 43057062-43057063	NM_007294.4(BRCA1):c.5266dupC (p.Gln1756Profs)	frameshift	rs80357906	0.0001803	Pathogenic	
SIGILO102	*BRCA1*	17: 43067606	NM_007294.4(BRCA1):c.5074+2T>C	splice donor	rs80358089	ND	Pathogenic	
SIGILO103	*BRCA1*	17: 43099808	NM_007294.4(BRCA1):c.514del (p.Gln172fs)	frameshift	rs80357872	0.000003977	Pathogenic	Described in Southern Italy (Sicily)[9]
SIGILO104	*BRCA1*	17: 43094839-43094842	NM_007294.4(BRCA1):c.689_692del (p.Glu230fs)	frameshift	rs886040308	ND	Pathogenic	
SIGILO105	*BRCA1*	17: 43094839-43094842	NM_007294.4(BRCA1):c.689_692del (p.Glu230fs)	frameshift	rs886040308	ND	Pathogenic	
SIGILO106	*BRCA1*	17: 43045766	NM_007294.4(BRCA1):c.5504G>A (p.Arg1835Gln)	missense	rs80356962	ND	Pathogenic	
SIGILO107	*BRCA1*	17: 43047665	NM_007294.4(BRCA1):c.5445G>A (p.Trp1815Ter)	nonsense	rs397509284	0.000003976	Pathogenic	
SIGILO108	*BRCA1*	17: 43067625	NM_007294.4(BRCA1):c.5057A>G (p.His1686Arg)	missense	rs730882166	ND	Pathogenic/ Likely-Pathogenic	
SIGILO109	*BRCA1*	17: 43092197-43092200	NM_007294.4(BRCA1):c.5057A>G (p.His1686Arg)	frameshift	rs80357701	ND	Pathogenic	
SIGILO110	*BRCA1*	17: 43057062-43057063	NM_007294.4(BRCA1):c.5266dup (p.Gln1756fs)	frameshift	rs80357906	0.0001803	Pathogenic	
SIGILO111	*BRCA1*	17: 43106457	NM_007294.4(BRCA1):c.211A>G (p.Arg71Gly)	missense	rs80357382	0.000004004	Pathogenic	
SIGILO112	*BRCA1*	17: 43057062-43057063	NM_007294.4(BRCA1):c.5266dupC (p.Gln1756Profs)	frameshift	rs80357906	0.0001803	Pathogenic	
SIGILO113	*BRCA1*	17: 43094839-43094842	NM_007294.4(BRCA1):c.689_692del (p.Glu230fs)	frameshift	rs886040308	ND	Pathogenic	
SIGILO114	*BRCA1*	17: 43092628-43092629	NM_007294.4(BRCA1):c.2901_2902dup (p.Pro968fs)	frameshift	rs398122670	ND	Pathogenic	
SIGILO115	*BRCA1*	17: 43106487	NM_007294.4(BRCA1):c.181T>G (p.Cys61Gly)	missense	rs28897672	0.00003190	pathogenic	
SIGILO116	*BRCA1*	17: 43094051	NM_007294.4(BRCA1):c.1480C>T (p.Gln494Ter)	nonsense	rs80357010	ND	Pathogenic	
SIGILO117	*BRCA1*	17: 43106457	NM_007294.4(BRCA1):c.211A>G (p.Arg71Gly)	missense	rs80357382	0.000004004	Pathogenic	

Table S4. continued

ID	Gee	Chr:Pos (GRCh38)	Variant	Type	dbsnp	gnomad	Clinvar/ ACMG	Comment
SIGILO118	*BRCA1*	17: 43067606	NM_007294.4(BRCA1):c.5074+2T>C	splice donor	rs80358089	ND	Pathogenic	
SIGILO119	*BRCA1*	17: 43092197-43092200	NM_007294.4(BRCA1):c.3331_3334del (p.Gln1111fs)	frameshift	rs80357701	ND	Pathogenic	
SIGILO120	*BRCA1*	17: 43106457	NM_007294.4(BRCA1):c.211A>G (p.Arg71Gly)	missense	rs80357382	0.000004004	Pathogenic	
SIGILO121	*BRCA1*	17: 43106457	NM_007294.4(BRCA1):c.211A>G (p.Arg71Gly)	missense	rs80357382	0.000004004	Pathogenic	
SIGILO122	*BRCA1*	17: 43063946-43063948	NM_007294.4(BRCA1):c.5078_5080del (p.Ala1693del)	inframe deletion	rs80358345	ND	Pathogenic/ Likely-Pathogenic	
SIGILO123	*BRCA1*	17: 43092197-43092200	NM_007294.4(BRCA1):c.3331_3334del (p.Gln1111fs)	frameshift	rs80357701	ND	Pathogenic	
SIGILO124	*BRCA1*	17: 43067606	NM_007294.4(BRCA1):c.5074+2T>C	splice donor	rs80358089	ND	Pathogenic	
SIGILO125	*BRCA1*	17: 43057062-43057063	NM_007294.4(BRCA1):c.5266dup (p.Gln1756fs)	frameshift	rs80357906	0.0001803	Pathogenic	
SIGILO126	*BRCA1*	17: 43094234	NM_007294.4(BRCA1):c.1297del (p.Ala433fs)	frameshift	rs80357794	ND	Pathogenic	
SIGILO127	*BRCA1*	17: 43057062-43057063	NM_007294.4(BRCA1):c.5266dup (p.Gln1756fs)	frameshift	rs80357906	0.0001803	Pathogenic	
SIGILO128	*BRCA1*	17: 43091496	NM_007294.4(BRCA1):c.4035del (p.Glu1346fs)	frameshift	rs80357711	0.00004248	Pathogenic	Eastern European founder variant[2,3]
SIGILO129	*BRCA1*	17: 43045758	NM_007294.4(BRCA1):c.5512del (p.Val1838fs)	frameshift	rs80357839	ND	Pathogenic	
SIGILO130	*BRCA1*	17: 43093844	NM_007294.4(BRCA1):c.1687C>T (p.Gln563Ter)	nonsense	rs80356898	0.00002795	Pathogenic	
SIGILO131	*BRCA1*	17: 43093845	NM_007294.4(BRCA1):c.1687C>T (p.Gln563Ter)	nonsense	rs80356899	0.00002796	Pathogenic	
SIGILO132	*BRCA1*	17: 43057062-43057063	NM_007294.4(BRCA1):c.5266dup (p.Gln1756fs)	frameshift	rs80357906	0.0001803	Pathogenic	
SIGILO133	*BRCA1*	17: 43067625	NM_007294.4(BRCA1):c.5057A>G (p.His1686Arg)	missense	rs730882166	ND	Pathogenic/ Likely-Pathogenic	
SIGILO134	*BRCA1*	17: 43067626	NM_007294.4(BRCA1):c.5057A>G (p.His1686Arg)	missense	rs730882167	ND	Pathogenic/ Likely-Pathogenic	
SIGILO135	*BRCA1*	17: 43057062-43057063	NM_007294.4(BRCA1):c.5266dup (p.Gln1756fs)	frameshift	rs80357906	0.0001803	Pathogenic	
SIGILO136	*BRCA1*	17:43091845	NM_007294.4(BRCA1):c.3686T>A (p.Leu1229Ter)	nonsense	rs2053543910	0.000001	Pathogenic	
SIGILO137	*BRCA1*	17: 43071175-43071178	NM_007294.4(BRCA1):c.4736_4739del (p.Pro1579fs)	frameshift	rs1555581017	ND	Pathogenic	
SIGILO138	*BRCA1*	17: 43092197-43092200	NM_007294.4(BRCA1):c.3331_3334del (p.Gln1111fs)	frameshift	rs80357701	ND	Pathogenic	
SIGILO139	*BRCA1*	17: 43092197-43092200	NM_007294.4(BRCA1):c.3331_3334del (p.Gln1111fs)	frameshift	rs80357701	ND	Pathogenic	
SIGILO140	*BRCA1*	17: 43094737-43094740	NM_007294.4(BRCA1):c.791_794del (p.Ser264fs)	frameshift	rs80357707	0.0000014	Pathogenic	
SIGILO141	*BRCA1*	17: 43093844	NM_007294.4(BRCA1):c.1687C>T (p.Gln563Ter)	nonsense	rs80356898	0.00002795	Pathogenic	
SIGILO142	*BRCA1*	17:43045743	NM_007294.4(BRCA1):c.5527G>C (p.Ala1843Pro)	missense	rs80357019	ND	Likely Pathogenic	
SIGILO143	*BRCA1*	17: 43057062-43057063	NM_007294.4(BRCA1):c.5266dup (p.Gln1756fs)	frameshift	rs80357906	0.0001803	Pathogenic	
SIGILO144	*BRCA1*	17:41275924-41277611	Heterozygous 1.7kb deletion involving exons 1 and 2 of the BRCA1 gene	Large Genomic Rearrangement	----	-----	Pathogenic	
SIGILO145	*BRCA1*	17: 43057052-43076614	NM_007294.3(BRCA1):c.4358-?_5277+?del	deletion exon 13		ND	Pathogenic	

Table S4. continued

ID	Gee	Chr:Pos (GRCh38)	Variant	Type	dbsnp	gnomad	Clinvar/ ACMG	Comment
SIGILO146	*BRCA1*	17: 43092197-43092200	NM_007294.4(BRCA1):c.3331_3334del (p.Gln1111fs)	frameshift	rs80357701	ND	Pathogenic	
SIGILO147	*BRCA1*	17:41.243.788	NM_007294.4:c.3756_3759del:p.(ser1253Argfs*10)	frameshift	rs80357868	0.000013	Pathogenic	
SIGILO148	*BRCA1*	17: 43093236	NM_007294.4(BRCA1):c.2295del (p.Ser766fs)	frameshift	rs1567796243	0.00002388	Pathogenic/ Likely-Pathogenic	
SIGILO149	*BRCA1*	17: 43057062-43057063	NM_007294.4(BRCA1):c.5266dup (p.Gln1756fs)	frameshift	rs80357906	0.0001803	Pathogenic	
SIGILO150	*BRCA1*	17: 43057062-43057063	NM_007294.4(BRCA1):c.5266dup (p.Gln1756fs)	frameshift	rs80357906	0.0001803	Pathogenic	
SIGILO151	*BRCA1*	17: 43093568-43093569 (	NM_007294.4(BRCA1):c.1962dup (p.Tyr655fs)	frameshift	rs1567797549	ND	Pathogenic	
SIGILO152	*BRCA1*	17: 43057062-43057063	NM_007294.4(BRCA1):c.5266dup (p.Gln1756fs)	frameshift	rs80357906	0.0001803	Pathogenic	
SIGILO153	*BRCA1*	17: 43104120	NM_007294.4(BRCA1):c.441+2T>A	splice donor	rs397509173	ND	Pathogenic/ Likely-Pathogenic	
SIGILO154	*BRCA1*	17: 43092197-43092200	NM_007294.4(BRCA1):c.3331_3334del (p.Gln1111fs)	frameshift	rs80357701	ND	Pathogenic	
SIGILO155	*BRCA1*	17: 43093844	NM_007294.4(BRCA1):c.1687C>T (p.Gln563Ter)	nonsense	rs80356898	0.00002795	Pathogenic	
SIGILO156	*BRCA1*	17: 43063903	NM_007294.4(BRCA1):c.5123C>A (p.Ala1708Glu)	missense	rs28897696	0.00040	Pathogenic	
SIGILO157	*BRCA1*	17: 43057062-43057063	NM_007294.4(BRCA1):c.5266dupC (p.Gln1756Profs)	frameshift	rs80357906	0.0001803	Pathogenic	
SIGILO158	*BRCA1*	17: 43106457	NM_007294.4(BRCA1):c.211A>G (p.Arg71Gly)	missense	rs80357382	0.000004004	Pathogenic	
SIGILO159	*BRCA1*	17: 43057062-43057063	NM_007294.4(BRCA1):c.5266dupC (p.Gln1756Profs)	frameshift	rs80357906	0.0001803	Pathogenic	
SIGILO160	*BRCA2*	13: 32337161-32337164	NM_000059.3(BRCA2):c.2808_2811del (p.Ala938Profs)	frameshift	rs80359351	0.000013	Pathogenic	
SIGILO161	*BRCA2*	13: 32337161-32337164	NM_000059.3(BRCA2):c.2808_2811del (p.Ala938Profs)	frameshift	rs80359351	0.000013	Pathogenic	
SIGILO162	*BRCA2*	13: 32338633-32338634	NM_000059.4(BRCA2):c.4284dup (p.Gln1429fs)	frameshift	rs80359439	0.00008	Pathogenic	
SIGILO163	*BRCA2*	13: 32338633-32338634	NM_000059.4(BRCA2):c.4284dup (p.Gln1429fs)	frameshift	rs80359439	0.000004091	Pathogenic	
SIGILO164	*BRCA2*	13: 32337161-32337164	NM_000059.3(BRCA2):c.2808_2811del (p.Ala938Profs)	frameshift	rs80359351	0.000013	Pathogenic	
SIGILO165	*BRCA2*	13: 32337185	NM_000059.4(BRCA2):c.2830A>T (p.Lys944Ter)	nonssense	rs80358533	0.000007	Pathogenic	
SIGILO166	*BRCA2*	13: 32338097-32338100	c.3744_3747del, p.(Ser1248Argfs*10).	frameshift	rs80359403	0.000007	Pathogenic	
SIGILO167	*BRCA2*	13: 32339320	NM_000059.4(BRCA2):c.4965C>A (p.Tyr1655Ter)	nonsense	rs80358721	0.000040	Pathogenic	
SIGILO168	*BRCA2*	13: 32326566	NM_000059.4(BRCA2):c.584C>G (p.Ser195Ter)	nonsense	rs886038053	ND	Pathogenic	
SIGILO169	*BRCA2*	13: 32326566	NM_000059.4(BRCA2):c.584C>G (p.Ser195Ter)	nonsense	rs886038053	ND	Pathogenic	
SIGILO170	*BRCA2*	13: 32354913	NM_000059.4(BRCA2):c.7060C>T (p.Gln2354Ter)	nonsense	rs80358936	0.000007	Pathogenic	
SIGILO171	*BRCA2*	13: 32379821-32379825	NM_000059.4(BRCA2):c.9026_9030del (p.Tyr3009fs)	frameshift	rs80359741	0.000002	Pathogenic	
SIGILO172	*BRCA2*	13: 32339555	NM_000059.4(BRCA2):c.5200G>T (p.Glu1734Ter)	nonsense	rs786202543	0.0000079	Pathogenic	
SIGILO173	*BRCA2*	13: 32340757-32340761	NM_000059.4(BRCA2):c.6405_6409del (p.Asn2135fs)	frameshift	rs80359584	0.000013	Pathogenic	
SIGILO174	*BRCA2*	13: 32340925	NM_000059.4(BRCA2):c.6405_6409del (p.Asn2135fs)	frameshift	rs1060502392	ND	Pathogenic	
SIGILO175	*BRCA2*	13: 32340925	NM_000059.4(BRCA2):c.6574del (p.Met2192fs)	frameshift	rs1060502392	ND	Pathogenic	
SIGILO176	*BRCA2*	13: 32354977	NM_000059.4(BRCA2):c.7124T>G (p.Leu2375Ter)	nonsense	rs886040687	ND	Pathogenic	

Table S4. continued

ID	Gee	Chr:Pos (GRCh38)	Variant	Type	dbsnp	gnomad	Clinvar/ ACMG	Comment
SIGILO177	*BRCA2*	13: 32355033	NM_000059.4(BRCA2):c.7180A>T (p.Arg2394Ter)	nonsense	rs80358946	0.000007	Pathogenic	
SIGILO178	*BRCA2*	13: 32357840-32357841	NM_000059.4(BRCA2):c.7717_7718del (p.Leu2573fs)	frameshift	rs2137567005	ND	Pathogenic	
SIGILO179	*BRCA2*	13: 32357840-32357841	NM_000059.4(BRCA2):c.7717_7718del (p.Leu2573fs)	frameshift	rs2137567005	ND	Pathogenic	
SIGILO180	*BRCA2*	13: 32379885-32379886	NM_000059.4(BRCA2):c.9097dup (p.Thr3033fs)	frameshift	rs397507419	0.000007	Pathogenic	
SIGILO181	*BRCA2*	13: 32379885-32379886	NM_000059.4(BRCA2):c.9097dup (p.Thr3033fs)	frameshift	rs397507419	0.000007	Pathogenic	
SIGILO182	*BRCA2*	13: 32339570-32339571	NM_000059.4(BRCA2):c.5216dup (p.Tyr1739Ter)	frameshift	rs886040578	ND	Pathogenic	
SIGILO183	*BRCA2*	13: 32344593 (GRCh38)	NM_000059.4(BRCA2):c.6879del (p.Phe2293fs)	frameshift	rs886040932	ND	Pathogenic	
SIGILO184	*BRCA2*	13: 32344593 (GRCh38)	NM_000059.4(BRCA2):c.6879del (p.Phe2293fs)	frameshift	rs886040932	ND	Pathogenic	
SIGILO185	*BRCA2*	13: 32344593 (GRCh38)	NM_000059.4(BRCA2):c.6879del (p.Phe2293fs)	frameshift	rs886040933	ND	Pathogenic	
SIGILO186	*BRCA2*	13: 32337161-32337164	NM_000059.3(BRCA2):c.2808_2811del (p.Ala938Profs)	frameshift	rs80359351	0.000013	Pathogenic	
SIGILO187	*BRCA2*	13: 32337161-32337164	NM_000059.3(BRCA2):c.2808_2811del (p.Ala938Profs)	frameshift	rs80359352	0.000014	Pathogenic	
SIGILO188	*BRCA2*	13: 32337161-32337164	NM_000059.3(BRCA2):c.2808_2811del (p.Ala938Profs)	frameshift	rs80359352	0.000014	Pathogenic	
SIGILO189	*BRCA2*	13: 32380043	NM_000059.4(BRCA2):c.9154C>T (p.Arg3052Trp)	missense	rs45580035	0.0000057	Pathogenic	
SIGILO190	*BRCA2*	13: 32326241	NM_000059.4(BRCA2):c.476-1G>A	splice acceptor	rs397507340	0.000007	Pathogenic/ Likely-Pathogenic	
SIGILO191	*BRCA2*	13: 32340037	NM_000059.4(BRCA2):c.5682C>G (p.Tyr1894T	nonssense	rs41293497	ND	Pathogenic	
SIGILO192	*BRCA2*	13: 32337161-32337164	NM_000059.3(BRCA2):c.2808_2811del (p.Ala938Profs)	frameshift	rs80359351	0.000013	Pathogenic	
SIGILO193	*BRCA2*	13: 32340000	NM_000059.4(BRCA2):c.5645C>A (p.Ser1882Ter)	nonsense	rs80358785	0.0000079	Pathogenic	
SIGILO194	*BRCA2*	13: 32340001	NM_000059.4(BRCA2):c.5645C>A (p.Ser1882Ter)	nonsense	rs80358786	0.0000080	Pathogenic	
SIGILO195	*BRCA2*	13: 32340001	NM_000059.4(BRCA2):c.5645C>A (p.Ser1882Ter)	nonsense	rs80358786	0.0000080	Pathogenic	
SIGILO196	*BRCA2*	13: 32326241	NM_000059.4(BRCA2):c.476-1G>A	splice aceptor	rs397507340	0.000007	Pathogenic/ Likely-Pathogenic	
SIGILO197	*BRCA2*	13: 32326241	NM_000059.4(BRCA2):c.476-1G>A	splice aceptor	rs397507340	0.000007	Pathogenic/ Likely-Pathogenic	
SIGILO198	*BRCA2*	13: 32340757-32340761	NM_000059.4(BRCA2):c.6405_6409del (p.Asn2135fs)	frameshift	rs80359584	0.000013	Pathogenic	
SIGILO199	*BRCA2*	13: 32340757-32340761	NM_000059.4(BRCA2):c.6405_6409del (p.Asn2135fs)	frameshift	rs80359584	0.000013	Pathogenic	
SIGILO200	*BRCA2*	13-32911527	NM_000059.4(BRCA2):c.3037del (p.Ser1013Glnfs*30)	frameshift	rs1566227658	ND	Likely Pathogenic	
SIGILO201	*BRCA2*	13-32911527	NM_000059.4(BRCA2):c.3037del (p.Ser1013Glnfs*30)	frameshift	rs1566227658	ND	Likely Pathogenic	
SIGILO202	*BRCA2*	13: 32340757-32340761	NM_000059.4(BRCA2):c.6405_6409del (p.Asn2135fs)	frameshift	rs80359584	0.000013	Pathogenic	
SIGILO203	*BRCA2*	13: 32340757-32340761	NM_000059.4(BRCA2):c.6405_6409del (p.Asn2135fs)	frameshift	rs80359584	0.000013	Pathogenic	
SIGILO204	*BRCA2*	13: 32339764-32339765	NM_000059.4(BRCA2):c.5410_5411del (p.Val1804fs)	frameshift	rs80359512	ND	Pathogenic	
SIGILO205	*BRCA2*	13: 32339764-32339765	NM_000059.4(BRCA2):c.5410_5411del (p.Val1804fs)	frameshift	rs80359512	ND	Pathogenic	

Table S4. continued

ID	Gee	Chr:Pos (GRCh38)	Variant	Type	dbsnp	gnomad	Clinvar/ ACMG	Comment
SIGILO206	*BRCA2*	13: 32340001	NM_000059.4(BRCA2):c.5645C>A (p.Ser1882Ter)	nonsense	rs80358786	0.0000080	Pathogenic	
SIGILO207	*BRCA2*	13: 32333283-32333284	NM_000059.4(BRCA2):c.1813dup (p.Ile605fs)	frameshift	rs80359306	0.00001293	Pathogenic	
SIGILO208	*BRCA2*	13: 32362694	NM_000059.4(BRCA2):c.7976+1G>A	splice donor	rs81002873	ND	Pathogenic	
SIGILO209	*BRCA2*	13: 32340037	NM_000059.4(BRCA2):c.5682C>G (p.Tyr1894T	nonsense	rs41293497	0.00003	Pathogenic	
SIGILO210	*BRCA2*	13: 32356472	NM_000059.4(BRCA2):c.7480C>T (p.Arg2494Ter)	nonsense	rs80358972	0.000013	Pathogenic	Reported in Korean populations (KOHBRA study)[10]
SIGILO211	*BRCA2*	13: 32362694	NM_000059.4(BRCA2):c.7976+1G>A	splice donor	rs81002873	ND	Pathogenic	
SIGILO212	*BRCA2*	13: 32376732	NM_000059.4(BRCA2):c.8695C>T (p.Gln2899Ter)	nonsense	rs397507411	0.0000007	Pathogenic	
SIGILO213	*BRCA2*	13: 32332815	NM_000059.4(BRCA2):c.1337T>A (p.Leu446Ter)	nonsense	rs1555281845	ND	Pathogenic	
SIGILO214	*BRCA2*	13: 32332815	NM_000059.4(BRCA2):c.1337T>A (p.Leu446Ter)	nonsense	rs1555281845	ND	Pathogenic	
SIGILO215	*BRCA2*	13:32376732	NM_000059.4(BRCA2):c.8695C>T (p.Gln2899Ter)	nonsense	rs397507411	0.0000007	Pathogenic	
SIGILO216	*BRCA2*	13:32376732	NM_000059.4(BRCA2):c.8695C>T (p.Gln2899Ter)	nonsense	rs397507411	0.0000007	Pathogenic	
SIGILO217	*BRCA2*	13: 32362574	NM_000059.4(BRCA2):c.7857G>A (p.Trp2619Ter)	nonsense	rs80359011	ND	Pathogenic	
SIGILO218	*BRCA2*	13: 32362574	NM_000059.4(BRCA2):c.7857G>A (p.Trp2619Ter)	nonsense	rs80359011	ND	Pathogenic	
SIGILO219	*BRCA2*	13: 32362574	NM_000059.4(BRCA2):c.7857G>A (p.Trp2619Ter)	nonsense	rs80359011	ND	Pathogenic	
SIGILO220	*BRCA2*	13: 32337161-32337164	NM_000059.3(BRCA2):c.2808_2811del (p.Ala938Profs)	frameshift	rs80359351	0.000013	Pathogenic	
SIGILO221	*BRCA2*	13: 32340837-32340840	NM_000059.4(BRCA2):c.6486_6489del (p.Lys2162fs)	frameshift	rs80359598	0.000020	Pathogenic	
SIGILO222	*BRCA2*	13: 32340837-32340840	NM_000059.4(BRCA2):c.6486_6489del (p.Lys2162fs)	frameshift	rs80359598	0.000020	Pathogenic	
SIGILO223	*BRCA2*	13: 32332815	NM_000059.4(BRCA2):c.1337T>A (p.Leu446Ter)	frameshift	rs1555281845	ND	Pathogenic	
SIGILO224	*BRCA2*	13: 32332771-32332772	NM_000059.4(BRCA2):c.1296_1297del (p.Asn433fs)	frameshift	rs80359276	ND	Pathogenic	
SIGILO225	*BRCA2*	13: 32339570-32339571	NM_000059.4(BRCA2):c.5216dup (p.Tyr1739Ter)	frameshift	rs886040578	ND	Pathogenic	
SIGILO226	*BRCA2*	13: 32339570-32339571	NM_000059.4(BRCA2):c.5216dup (p.Tyr1739Ter)	frameshift	rs886040578	ND	Pathogenic	
SIGILO227	*BRCA2*	13: 32357862	NM_000059.4(BRCA2):c.7738C>T (p.Gln2580Ter)	nonsense	rs80358999	ND	Pathogenic	
SIGILO228	*BRCA2*	13: 32337161-32337164	NM_000059.3(BRCA2):c.2808_2811del (p.Ala938Profs)	frameshift	rs80359351	0.000013	Pathogenic	
SIGILO229	*BRCA2*	13: 32316462	NM_000059.4(BRCA2):c.2T>G (p.Met1Arg)	initiator codon variant missense	rs80358547	0.000002	Pathogenic	
SIGILO230	*BRCA2*	13: 32326498	NM_000059.4(BRCA2):c.517-1G>A	splice acceptor	rs81002849	ND	Pathogenic	
SIGILO231	*BRCA2*	13: 32337161-32337164	NM_000059.3(BRCA2):c.2808_2811del (p.Ala938Profs)	frameshift	rs80359351	0.000013	Pathogenic	
SIGILO232	*BRCA2*	13: 32337161-32337164	NM_000059.3(BRCA2):c.2808_2811del (p.Ala938Profs)	frameshift	rs80359351	0.000013	Pathogenic	
SIGILO233	*BRCA2*	13: 32340217-32340218	NM_000059.4(BRCA2):c.5862_5863del (p.Ser1955fs)	frameshift	rs786202700	ND	Pathogenic	
SIGILO234	*BRCA2*	13: 32316529	NM_000059.4(BRCA2):c.67+2T>A	splice donor	rs81002885	0.000004	Pathogenic	

Table S4. continued

ID	Gee	Chr:Pos (GRCh38)	Variant	Type	dbsnp	gnomad	Clinvar/ ACMG	Comment
SIGILO235	*BRCA2*	13: 32316529	NM_000059.4(BRCA2):c.67+2T>A	splice donor	rs81002885	0.000004	Pathogenic	
SIGILO236	*BRCA2*	13: 32337161-32337164	NM_000059.3(BRCA2):c.2808_2811del (p.Ala938Profs)	frameshift	rs80359351	0.000013	Pathogenic	
SIGILO237	*BRCA2*	13: 32379440	NM_000059.4(BRCA2):c.8878C>T (p.Gln2960Ter)	nonssense	rs80359140	0.000006	Pathogenic	
SIGILO238	*BRCA2*	13: 32379440	NM_000059.4(BRCA2):c.8878C>T (p.Gln2960Ter)	nonssense	rs80359140	0.000006	Pathogenic	
SIGILO239	*BRCA2*	13: 32337161-32337164	NM_000059.3(BRCA2):c.2808_2811del (p.Ala938Profs)	frameshift	rs80359351	0.000013	Pathogenic	
SIGILO240	*BRCA2*	13: 32337161-32337164	NM_000059.3(BRCA2):c.2808_2811del (p.Ala938Profs)	frameshift	rs80359351	0.000013	Pathogenic	
SIGILO241	*BRCA2*	13: 32337618-32337619	NM_000059.4(BRCA2):c.3264dup (p.Gln1089fs)	frameshift	rs80359380	0.000007	Pathogenic	
SIGILO242	*BRCA2*	13: 32337161-32337164	NM_000059.3(BRCA2):c.2808_2811del (p.Ala938Profs)	frameshift	rs80359351	0.000013	Pathogenic	
SIGILO243	*BRCA2*	13: 32337161-32337164	NM_000059.3(BRCA2):c.2808_2811del (p.Ala938Profs)	frameshift	rs80359351	0.000013	Pathogenic	
SIGILO244	*BRCA2*	13: 32338486-32338487	NM_000059.4(BRCA2):c.4131_4132insTGAGGA (p.Thr1378Ter)	nonssense	rs80359429	0.000002	Pathogenic	
SIGILO245	*BRCA2*	13: 32326498	NM_000059.4(BRCA2):c.517-1G>A	splice donor	rs81002849	ND	Pathogenic	
SIGILO246	*BRCA2*	13: 32326498	NM_000059.4(BRCA2):c.517-1G>A	splice donor	rs81002849	ND	Pathogenic	
SIGILO247	*BRCA2*	13: 32337618-32337619	NM_000059.4(BRCA2):c.3264dup (p.Gln1089fs)	frameshift	rs80359380	0.000007	Pathogenic	
SIGILO248	*BRCA2*	13: 32340367-32340370	NM_000059.4(BRCA2):c.6014_6017del (p.Asp2005fs)	frameshift	rs1057518635	ND	Pathogenic	
SIGILO249	*BRCA2*	13: 32340367-32340370	NM_000059.4(BRCA2):c.6014_6017del (p.Asp2005fs)	frameshift	rs1057518635	ND	Pathogenic	
SIGILO250	*BRCA2*	13: 32356550	NM_000059.4(BRCA2):c.7558C>T (p.Arg2520Ter)	nonssense	rs80358981	0.0000071	Pathogenic	
SIGILO251	*BRCA2*	13: 32340837-32340840	NM_000059.4(BRCA2):c.6486_6489del (p.Lys2162fs)	frameshift	rs80359598	0.000020	Pathogenic	
SIGILO252	*BRCA2*	13: 32337161-32337164	NM_000059.3(BRCA2):c.2808_2811del (p.Ala938Profs)	frameshift	rs80359351	0.000013	Pathogenic	
SIGILO253	*BRCA2*	13: 32336731	NM_000059.4(BRCA2):c.2376C>G (p.Tyr792Ter)	nonssense	s80358503	ND	Pathogenic	
SIGILO254	*BRCA2*		Duplication exon 5-11 BRCA2	large rearrangem ent			Pathogenic	First described in Israeli Arab Christian population[11]
SIGILO255	*BRCA2*	13: 3233682	NM_000059.4(BRCA2):c.2466del (p.Cys822fs)	frameshift	rs2548524019	ND	Pathogenic	
SIGILO256	*BRCA2*	13: 32340037	NM_000059.4(BRCA2):c.5682C>G (p.Tyr1894T	nonssense	rs41293497	0.00003	Pathogenic	
SIGILO257	*BRCA2*	13: 32326498	NM_000059.4(BRCA2):c.517-1G>A	splice acceptor	rs81002849	ND	Pathogenic	
SIGILO258	*BRCA2*	13: 32337161-32337164	NM_000059.3(BRCA2):c.2808_2811del (p.Ala938Profs)	frameshift	rs80359351	0.000013	Pathogenic	
SIGILO259	*BRCA2*	13: 32341011	NM_000059.4(BRCA2):c.6656C>G (p.Ser2219Ter)	nonsense	rs80358893	ND	Pathogenic	
SIGILO260	*BRCA2*	13: 32341011	NM_000059.4(BRCA2):c.6656C>G (p.Ser2219Ter)	nonsense	rs80358893	ND	Pathogenic	
SIGILO261	*BRCA2*	13: 32340846-32340849	NM_000059.4(BRCA2):c.6491_6494del (p.Gln2164fs)	frameshift	rs397507862	0.000002	Pathogenic	
SIGILO262	*BRCA2*	13: 32340846-32340849	NM_000059.4(BRCA2):c.6491_6494del (p.Gln2164fs)	frameshift	rs397507862	0.000002	Pathogenic	

Table S4. continued

ID	Gee	Chr:Pos (GRCh38)	Variant	Type	dbsnp	gnomad	Clinvar/ ACMG	Comment
SIGILO263	*BRCA2*	13: 32371052-32371053	NM_000059.4(BRCA2):c.8585dup (p.Glu2863fs)	frameshift	rs80359720	0.0000007	Pathogenic	
SIGILO264	*BRCA2*	13: 32339808	NM_000059.4(BRCA2):c.5453C>A (p.Ser1818Ter)	nonssense	rs1566232471	0.000001	Pathogenic	
SIGILO265	*BRCA2*	13: 32340837-32340840	NM_000059.4(BRCA2):c.6486_6489del (p.Lys2162fs)	frameshift	rs80359598	0.000020	Pathogenic	
SIGILO266	*BRCA2*	13: 32340837-32340840	NM_000059.4(BRCA2):c.6486_6489del (p.Lys2162fs)	frameshift	rs80359598	0.000020	Pathogenic	
SIGILO267	*BRCA2*	13: 32340837-32340840	NM_000059.4(BRCA2):c.6486_6489del (p.Lys2162fs)	frameshift	rs80359598	0.000020	Pathogenic	
SIGILO268	*BRCA2*	13: 32340722-32340723	NM_000059.4(BRCA2):c.6373dup (p.Thr2125fs)	frameshift	rs80359577	ND	Pathogenic	
SIGILO269	*TP53*	17: 7670699	NM_000546.6(TP53):c.1010G>A (p.Arg337His)	missense	rs121912664	0.000013	Pathogenic/ Likely-Pathogenic Pathogenic, low penetrance	Brazilian founder variant[12–14]
SIGILO270	*TP53*	17: 7674893	NM_000546.6(TP53):c.638G>A (p.Arg213Gln)	missense	rs587778720	0.0000029	Pathogenic/ Likely-Pathogenic	
SIGILO271	*TP53*	17: 7670699	NM_000546.6(TP53):c.1010G>A (p.Arg337His)	missense	rs121912664	0.000013	Pathogenic/ Likely-Pathogenic /Pathogenic, low penetrance	
SIGILO272	*TP53*	17: 7670699	NM_000546.6(TP53):c.1010G>A (p.Arg337His)	missense	rs121912664	0.000013	Pathogenic/ Likely-Pathogenic /Pathogenic, low penetrance	
SIGILO273	*TP53*	17: 7670699	NM_000546.6(TP53):c.1010G>A (p.Arg337His)	missense	rs121912664	0.000013	Pathogenic/ Likely-Pathogenic /Pathogenic, low penetrance	
SIGILO274	*TP53*	17: 7670699	NM_000546.6(TP53):c.1010G>A (p.Arg337His)	missense	rs121912664	0.000013	Pathogenic/Likely Pathogenic/Pathogenic , low penetrance	
SIGILO275	*TP53*	17: 7670699	NM_000546.6(TP53):c.1010G>A (p.Arg337His)	missense	rs121912664	0.000013	Pathogenic/Likely Pathogenic/Pathogenic , low penetrance	
SIGILO276	*TP53*	17: 7670699	NM_000546.6(TP53):c.1010G>A (p.Arg337His)	missense	rs121912664	0.000013	Pathogenic/Likely Pathogenic/Pathogenic , low penetrance	
SIGILO277	*TP53*	17: 7670699	NM_000546.6(TP53):c.1010G>A (p.Arg337His)	missense	rs121912664	0.000013	Pathogenic/Likely Pathogenic/Pathogenic , low penetrance	
SIGILO278	*TP53*	17: 7670699	NM_000546.6(TP53):c.1010G>A (p.Arg337His)	missense	rs121912664	0.000013	Pathogenic/Likely Pathogenic/Pathogenic , low penetrance	
SIGILO279	*TP53*	17: 7670699	NM_000546.6(TP53):c.1010G>A (p.Arg337His)	missense	rs121912664	0.000013	Pathogenic/Likely Pathogenic/Pathogenic , low penetrance	

Table S4. continued

ID	Gee	Chr:Pos (GRCh38)	Variant	Type	dbsnp	gnomad	Clinvar/ ACMG	Comment
SIGILO280	*TP53*	17: 7670699	NM_000546.6(TP53):c.1010G>A (p.Arg337His)	missense	rs121912664	0.000013	Pathogenic/Likely Pathogenic/Pathogenic, low penetrance	
SIGILO281	*TP53*	17: 7670699	NM_000546.6(TP53):c.1010G>A (p.Arg337His)	missense	rs121912664	0.000013	Pathogenic/Likely Pathogenic/Pathogenic, low penetrance	
SIGILO282	*TP53*	17: 7670699	NM_000546.6(TP53):c.1010G>A (p.Arg337His)	missense	rs121912664	0.000013	Pathogenic/Likely Pathogenic/Pathogenic, low penetrance	
SIGILO283	*TP53*	17: 7670699	NM_000546.6(TP53):c.1010G>A (p.Arg337His)	missense	rs121912664	0.000013	Pathogenic/Likely Pathogenic/Pathogenic, low penetrance	
SIGILO284	*TP53*	17: 7670699	NM_000546.6(TP53):c.1010G>A (p.Arg337His)	missense	rs121912664	0.000013	Pathogenic/Likely Pathogenic/Pathogenic, low penetrance	
SIGILO285	*TP53*	17: 7670699	NM_000546.6(TP53):c.1010G>A (p.Arg337His)	missense	rs121912664	0.000013	Pathogenic/Likely Pathogenic/Pathogenic, low penetrance	
SIGILO286	*TP53*	17: 7670699	NM_000546.6(TP53):c.1010G>A (p.Arg337His)	missense	rs121912664	0.000013	Pathogenic/Likely Pathogenic/Pathogenic, low penetrance	
SIGILO287	*TP53*	17: 7670699	NM_000546.6(TP53):c.1010G>A (p.Arg337His)	missense	rs121912664	0.000013	Pathogenic/Likely Pathogenic/Pathogenic, low penetrance	
SIGILO288	*TP53*	17: 7670699	NM_000546.6(TP53):c.1010G>A (p.Arg337His)	missense	rs121912664	0.000013	Pathogenic/Likely Pathogenic/Pathogenic, low penetrance	
SIGILO289	*TP53*	17: 7670699	NM_000546.6(TP53):c.1010G>A (p.Arg337His)	missense	rs121912664	0.000013	Pathogenic/Likely Pathogenic/Pathogenic, low penetrance	
SIGILO290	*TP53*	17: 7670699	NM_000546.6(TP53):c.1010G>A (p.Arg337His)	missense	rs121912664	0.000013	Pathogenic/Likely Pathogenic/Pathogenic, low penetrance	
SIGILO291	*TP53*	17: 7670699	NM_000546.6(TP53):c.1010G>A (p.Arg337His)	missense	rs121912664	0.000013	Pathogenic/Likely Pathogenic/Pathogenic, low penetrance	
SIGILO292	*TP53*	17: 7670699	NM_000546.6(TP53):c.1010G>A (p.Arg337His)	missense	rs121912664	0.000013	Pathogenic/Likely Pathogenic/Pathogenic, low penetrance	
SIGILO293	*TP53*	17: 7670699	NM_000546.6(TP53):c.1010G>A (p.Arg337His)	missense	rs121912664	0.000013	Pathogenic/Likely Pathogenic/Pathogenic, low penetrance	
SIGILO294	*TP53*	17: 7670699	NM_000546.6(TP53):c.1010G>A (p.Arg337His)	missense	rs121912664	0.000013	Pathogenic/Likely Pathogenic/Pathogenic, low penetrance	
SIGILO295	*TP53*	17: 7670699	NM_000546.6(TP53):c.1010G>A (p.Arg337His)	missense	rs121912664	0.000013	Pathogenic/Likely Pathogenic/Pathogenic, low penetrance	
SIGILO296	*TP53*	17: 7670699	NM_000546.6(TP53):c.1010G>A (p.Arg337His)	missense	rs121912664	0.000013	Pathogenic/Likely Pathogenic/Pathogenic, low penetrance	

Table S4. continued

ID	Gee	Chr:Pos (GRCh38)	Variant	Type	dbsnp	gnomad	Clinvar/ ACMG	Comment
SIGILO297	*TP53*	17: 7670699	NM_000546.6(TP53):c.1010G>A (p.Arg337His)	missense	rs121912664	0.000013	Pathogenic/Likely Pathogenic/Pathogenic, low penetrance	
SIGILO298	*TP53*	17: 7670699	NM_000546.6(TP53):c.1010G>A (p.Arg337His)	missense	rs121912664	0.000013	Pathogenic/Likely Pathogenic/Pathogenic, low penetrance	
SIGILO299	*TP53*	17: 7670699	NM_000546.6(TP53):c.1010G>A (p.Arg337His)	missense	rs121912664	0.000013	Pathogenic/Likely Pathogenic/Pathogenic, low penetrance	
SIGILO300	*TP53*	17: 7670699	NM_000546.6(TP53):c.1010G>A (p.Arg337His)	missense	rs121912664	0.000013	Pathogenic/Likely Pathogenic/Pathogenic, low penetrance	
SIGILO301	*TP53*	17: 7670699	NM_000546.6(TP53):c.1010G>A (p.Arg337His)	missense	rs121912664	0.000013	Pathogenic/Likely Pathogenic/Pathogenic, low penetrance	
SIGILO302	*TP53*	17: 7670699	NM_000546.6(TP53):c.1010G>A (p.Arg337His)	missense	rs121912664	0.000013	Pathogenic/Likely Pathogenic/Pathogenic, low penetrance	
SIGILO303	*TP53*	17: 7670657	NM_000546.6(TP53):c.1052del (p.Lys351fs)	frameshift	rs2150994507	ND	Likely Pathogenic	
SIGILO304	*TP53*	17: 7675234	NM_000546.6(TP53):c.378C>G (p.Tyr126Ter)	nonsense	rs1567554500	0.0000007	Pathogenic/Likely Pathogenic	
SIGILO305	*TP53*	17: 7675095	NM_000546.6(TP53):c.517G>A (p.Val173Met)	missense	rs876660754	ND	Pathogenic	
SIGILO306	*TP53*	17: 7675088	NM_000546.6(TP53):c.524G>A (p.Arg175His)	missense	rs28934578	0.000007	Pathogenic	
SIGILO307	*TP53*	17: 7670699	NM_000546.6(TP53):c.1010G>A (p.Arg337His)	missense	rs121912664	0.000013	Pathogenic/Likely Pathogenic/Pathogenic, low penetrance	
SIGILO308	*TP53*	17: 7675994	NM_000546.6(TP53):c.375G>A (p.Thr125=)	synonymous	rs55863639	0.000002	Pathogenic/Likely Pathogenic	
SIGILO309	*TP53*	17: 7674245	NM_000546.6(TP53):c.718A>G (p.Ser240Gly)	missense	rs1567549584	ND	Pathogenic/Likely Pathogenic	
SIGILO310	*TP53*	17: 7670699	NM_000546.6(TP53):c.1010G>A (p.Arg337His)	missense	rs121912664	0.000013	Pathogenic/Likely Pathogenic/Pathogenic, low penetrance	
SIGILO311	*TP53*	17: 7670699	NM_000546.6(TP53):c.1010G>A (p.Arg337His)	missense	rs121912664	0.000013	Pathogenic/Likely Pathogenic/Pathogenic, low penetrance	
SIGILO312	*TP53*	17: 7670699	NM_000546.6(TP53):c.1010G>A (p.Arg337His)	missense	rs121912664	0.000013	Pathogenic/Likely Pathogenic/Pathogenic, low penetrance	
SIGILO313	*TP53*	17: 7670699	NM_000546.6(TP53):c.1010G>A (p.Arg337His)	missense	rs121912664	0.000013	Pathogenic/Likely Pathogenic/Pathogenic, low penetrance	
SIGILO314	*TP53*	17: 7670699	NM_000546.6(TP53):c.1010G>A (p.Arg337His)	missense	rs121912664	0.000013	Pathogenic/Likely Pathogenic/Pathogenic, low penetrance	
SIGILO315	*TP53*	17: 7670699	NM_000546.6(TP53):c.1010G>A (p.Arg337His)	missense	rs121912664	0.000013	Pathogenic/Likely Pathogenic/Pathogenic, low penetrance	
SIGILO316	*TP53*	17: 7670699	NM_000546.6(TP53):c.1010G>A (p.Arg337His)	missense	rs121912664	0.000013	Pathogenic/Likely Pathogenic/Pathogenic, low penetrance	

Table S4. continued

ID	Gee	Chr:Pos (GRCh38)	Variant	Type	dbsnp	gnomad	Clinvar/ ACMG	Comment
SIGILO317	*TP53*	17: 7670699	NM_000546.6(TP53):c.1010G>A (p.Arg337His)	missense	rs121912664	0.000013	Pathogenic/Likely Pathogenic/Pathogenic, low penetrance	
SIGILO318	*TP53*	17: 7670699	NM_000546.6(TP53):c.1010G>A (p.Arg337His)	missense	rs121912664	0.000013	Pathogenic/Likely Pathogenic/Pathogenic, low penetrance	
SIGILO319	*TP53*	17: 7674230	NM_000546.6(TP53):c.733G>A (p.Gly245Ser)	missense	rs28934575	0.000007	Pathogenic	
SIGILO320	*TP53*	17: 7674230	NM_000546.6(TP53):c.733G>A (p.Gly245Ser)	missense	rs28934575	0.000007	Pathogenic	
SIGILO321	*TP53*	17: 7674230	NM_000546.6(TP53):c.733G>A (p.Gly245Ser)	missense	rs28934575	0.000007	Pathogenic	
SIGILO322	*TP53*	17: 7670699	NM_000546.6(TP53):c.1010G>A (p.Arg337His)	missense	rs121912664	0.000013	Pathogenic/Likely Pathogenic/Pathogenic, low penetrance	
SIGILO323	*TP53*	17: 7670699	NM_000546.6(TP53):c.1010G>A (p.Arg337His)	missense	rs121912664	0.000013	Pathogenic/Likely Pathogenic/Pathogenic, low penetrance	
SIGILO324	*TP53*	17: 7670699	NM_000546.6(TP53):c.1010G>A (p.Arg337His)	missense	rs121912664	0.000013	Pathogenic/Likely Pathogenic/Pathogenic, low penetrance	
SIGILO325	*TP53*	17: 7670657	NM_000546.6(TP53):c.1052del (p.Lys351fs)	frameshift	rs2150994507	ND	Likely Pathogenic	
SIGILO326	*TP53*	17: 7670699	NM_000546.6(TP53):c.1010G>A (p.Arg337His)	missense	rs121912664	0.000013	Pathogenic/Likely Pathogenic/Pathogenic, low penetrance	
SIGILO327	*TP53*	17: 7670699	NM_000546.6(TP53):c.1010G>A (p.Arg337His)	missense	rs121912664	0.000013	Pathogenic/Likely Pathogenic/Pathogenic, low penetrance	
SIGILO328	*TP53*	17: 7670699	NM_000546.6(TP53):c.1010G>A (p.Arg337His)	missense	rs121912664	0.000013	Pathogenic/Likely Pathogenic/Pathogenic, low penetrance	
SIGILO329	*TP53*	17: 7670699	NM_000546.6(TP53):c.1010G>A (p.Arg337His)	missense	rs121912664	0.000013	Pathogenic/Likely Pathogenic/Pathogenic, low penetrance	
SIGILO330	*TP53*	17: 7670699	NM_000546.6(TP53):c.1010G>A (p.Arg337His)	missense	rs121912664	0.000013	Pathogenic/Likely Pathogenic/Pathogenic, low penetrance	
SIGILO331	*TP53*	17: 7670699	NM_000546.6(TP53):c.1010G>A (p.Arg337His)	missense	rs121912664	0.000013	Pathogenic/Likely Pathogenic/Pathogenic, low penetrance	
SIGILO332	*TP53*	17: 7670699	NM_000546.6(TP53):c.1010G>A (p.Arg337His)	missense	rs121912664	0.000013	Pathogenic/Likely Pathogenic/Pathogenic, low penetrance	
SIGILO333	*TP53*	17: 7670699	NM_000546.6(TP53):c.1010G>A (p.Arg337His)	missense	rs121912664	0.000013	Pathogenic/Likely Pathogenic/Pathogenic, low penetrance	
SIGILO334	*TP53*	17: 7670699	NM_000546.6(TP53):c.1010G>A (p.Arg337His)	missense	rs121912664	0.000013	Pathogenic/Likely Pathogenic/Pathogenic, low penetrance	
SIGILO335	*PALB2*	16: 23626273	NM_024675.4(PALB2):c.2711G>A (p.Trp904Ter)	nonsense	rs1060502726	0.0000021	Pathogenic	

Table S4. continued

ID	Gee	Chr:Pos (GRCh38)	Variant	Type	dbsnp	gnomad	Clinvar/ ACMG	Comment
SIGILO336	*PALB2*	16: 23635354	NM_024675.4(PALB2):c.1192del (p.Val398fs)	frameshift	rs1555461407	0.000002	Pathogenic	
SIGILO337	*PALB2*	16: 23635006-23635007	NM_024675.4(PALB2):c.1539dup (p.Gly514fs)	frameshift	rs1567220784	ND	Pathogenic	
SIGILO338	*PALB2*	16: 23629897	NM_024675.4(PALB2):c.2257C>T (p.Arg753Ter)	nonsense	rs180177110	0.000007	Pathogenic	
SIGILO339	*PALB2*	16: 23626273	NM_024675.4(PALB2):c.2711G>A (p.Trp904Ter)	nonsense	rs1060502726	0.0000021	Pathogenic	
SIGILO340	*PALB2*	16: 23635831	NM_024675.4(PALB2):c.715del (p.Arg239fs)	frameshift	rs1567222476	0.00001	Pathogenic	Recurrent in Japanese population[15]
SIGILO341	*PALB2*	16: 23641115	NM_024675.4(PALB2):c.43G>T (p.Glu15Ter)	nonsense	rs730881884	0.000013		
SIGILO342	*PALB2*	16: 23603512-23603513	NM_024675.4(PALB2):c.3507_3508del (p.His1170fs)	frameshift	rs587776428	0.000007	Pathogenic	
SIGILO343	*PALB2*	16: 23603512-23603513	NM_024675.4(PALB2):c.3507_3508del (p.His1170fs)	frameshift	rs587776428	0.000007	Pathogenic	
SIGILO344	*PALB2*	16: 23603512-23603514	NM_024675.4(PALB2):c.3507_3508del (p.His1170fs)	frameshift	rs587776429	0.000008	Pathogenic	
SIGILO345	*PALB2*	16: 23603512-23603515	NM_024675.4(PALB2):c.3507_3508del (p.His1170fs)	frameshift	rs587776430	0.000009	Pathogenic	
SIGILO346	*PALB2*	16: 23641115	NM_024675.4(PALB2):c.43G>T (p.Glu15Ter)	nonsense	rs730881884	0.000013		
SIGILO347	*PALB2*	16: 23626273	NM_024675.4(PALB2):c.2711G>A (p.Trp904Ter)	nonsense	rs1060502726	0.0000021	Pathogenic	
SIGILO348	*PALB2*	16: 23635787-23635788	NM_024675.4(PALB2):c.758dup (p.Ser254fs)	frameshift	rs515726126	0.0000128	Pathogenic/Likely Pathogenic	
SIGILO349	*PALB2*	16: 23635787-23635788	NM_024675.4(PALB2):c.758dup (p.Ser254fs)	frameshift	rs515726126	0.0000128	Pathogenic/Likely Pathogenic	
SIGILO350	*PALB2*	16: 23635354	NM_024675.4(PALB2):c.1192del (p.Val398fs)	frameshift	rs1555461407	0.000002	Pathogenic	
SIGILO351	*PALB2*	16: 23635355	NM_024675.4(PALB2):c.1192del (p.Val398fs)	frameshift	rs1555461408	0.000003	Pathogenic	
SIGILO352	*PALB2*	16: 23636320	NM_024675.4(PALB2):c.226del (p.Ile76fs	frameshift	rs587782443	0.0000007	Pathogenic	
SIGILO353	*PALB2*	16:23.640.565-23.640.566	NG_007406.1:g.(5249_8228)_(17155_19960)del	deletion-EX6	---	----	Pathogenic	
SIGILO354	*PALB2*	16: 23641115	NM_024675.4(PALB2):c.43G>T (p.Glu15Ter)	nonsense	rs730881884	0.000013	Pathogenic	
SIGILO355	*PALB2*	16: 23629897	NM_024675.4(PALB2):c.2257C>T (p.Arg753Ter)	nonsense	rs180177110	0.000007	Pathogenic	
SIGILO356	*PALB2*	16: 23636036-23636037	NM_024675.4(PALB2):c.509_510del (p.Arg170fs)	frameshift	rs515726123	0.0000193	Pathogenic	
SIGILO357	*PALB2*	16: 23630137-23630138	NM_024675.4(PALB2):c.2016dup (p.Glu673fs)	frameshift	rs1966861906	ND	Pathogenic	
SIGILO358	*PALB2*	16: 23629897	NM_024675.4(PALB2):c.2257C>T (p.Arg753Ter)	nonsense	rs180177110	0.000007	Pathogenic	
SIGILO359	*PALB2*	16: 23635306	NM_024675.4(PALB2):c.1240C>T (p.Arg414Ter)	nonsense	rs180177100	0.000013	Pathogenic	
SIGILO360	*PALB2*	16:23.640.565-23.640.566	NG_007406.1:g.(5249_8228)_(17155_19960)del	deletion-EX6			Pathogenic	
SIGILO361	*PALB2*	16: 23636191	NM_024675.4(PALB2):c.355del (p.Gln119fs)	frameshift	rs1060502760	ND	Pathogenic	
SIGILO362	*PALB2*	16: 23637886-23637889	NM_024675.4(PALB2):c.172_175del (p.Gln60fs)	frameshift	rs180177143	0.000027	Pathogenic	
SIGILO363	*PALB2*	16: 23635306	NM_024675.4(PALB2):c.1240C>T (p.Arg414Ter)	nonsense	rs180177100	0.000013	Pathogenic	
SIGILO364	*PALB2*	16: 23635306	NM_024675.4(PALB2):c.1240C>T (p.Arg414Ter	nonsense	rs180177100	0.000013	Pathogenic	
SIGILO365	*ATM*	11: 108327665-108327668	NM_000051.4(ATM):c.7000_7003del (p.Tyr2334fs)	frameshift	rs786203421	0.0000021	Pathogenic/Likely Pathogenic	
SIGILO366	*ATM*	11: 108271147	NM_000051.4(ATM):c.2921+1G>A	splice donor	rs587781558	ND	Pathogenic	
SIGILO367	*ATM*	11: 108271147	NM_000051.4(ATM):c.2921+1G>A	splice donor	rs587781558	ND	Pathogenic	
SIGILO368	*ATM*	11: 108327665-108327668	NM_000051.4(ATM):c.7000_7003del (p.Tyr2334fs)	frameshift	rs786203421	0.0000021	Pathogenic/Likely Pathogenic	

Table S4. continued

ID	Gee	Chr:Pos (GRCh38)	Variant	Type	dbsnp	gnomad	Clinvar/ ACMG	Comment
SIGILO369	*ATM*	11: 108327665-108327668	NM_000051.4(ATM):c.7000_7003del (p.Tyr2334fs)	frameshift	rs786203421	0.0000021	Pathogenic/Likely Pathogenic	
SIGILO370	*ATM*	11: 108331442-108331445	NM_000051.4(ATM):c.7517_7520del	splice acceptor	rs587781905	ND	Pathogenic	
SIGILO371	*ATM*	11: 108284474	NM_000051.4(ATM):c.3993+1G>A	splice donor	rs200196781	0.000012	Pathogenic/Likely Pathogenic	
SIGILO372	*ATM*	11: 108244066	NM_000051.4(ATM):c.610G>T (p.Gly204Ter)	nonsense	rs147915571	0.0000821	Pathogenic	
SIGILO373	*ATM*	11: 108304822	NM_000051.4(ATM):c.5644C>T (p.Arg1882Ter)	nonsense	rs786204433	0.0000050	Pathogenic	
SIGILO374	*ATM*	11: 108321410	NM_000051.4(ATM):c.6562C>G (p.Leu2188Val)	missense	rs1060501682	0.0000021	Pathogenic	
SIGILO375	*ATM*	11: 108310316	NM_000051.4(ATM):c.5918+1G>T	splice donor	rs1591746871	0.0000014	Likely Pathogenic	
SIGILO376	*ATM*	11: 108284281	NM_000051.4(ATM):c.3802del (p.Glu1267_Val1268insTer)	nonsense	rs587779834	0.0000921	Pathogenic	
SIGILO377	*ATM*	11: 108244873	NM_000051.4(ATM):c.748C>T (p.Arg250Ter)	nonsense	rs772821016	ND	Pathogenic	
SIGILO378	*ATM*	11: 108284281	NM_000051.4(ATM):c.3802del (p.Glu1267_Val1268insTer)	nonsense	rs587779834	0.0000921	Pathogenic	
SIGILO379	*ATM*	11: 108335080	NM_000051.4(ATM):c.8122G>A (p.Asp2708Asn)	missense	rs587782719	0.000007	Pathogenic	
SIGILO380	*ATM*	11: 108331877	NM_000051.4(ATM):c.7630-2A>C	splice acceptor	rs587779866	0.000013	Pathogenic	
SIGILO381	*ATM*	11: 108249102	NM_000051.4(ATM):c.1235G>A (p.Trp412Ter)	nonsense	rs587779813	0.000007	Pathogenic	
SIGILO382	*ATM*	11: 108325416	NM_000051.4(ATM):c.6679C>T (p.Arg2227Cys)	missense	rs564652222	0.0000064	Pathogenic	
SIGILO383	*ATM*	11: 108332886	NM_000051.4(ATM):c.7913G>A (p.Trp2638Ter)	nonsense	rs377349459	0.0000029	Pathogenic/Likely Pathogenic	
SIGILO384	*ATM*	11: 108304822	NM_000051.4(ATM):c.5644C>T (p.Arg1882Ter)	nonsense	rs786204433	0.0000050	Pathogenic	
SIGILO385	*ATM*	11: 108310158	NM_000051.4(ATM):c.5763-2A>T	splice acceptor	rs876659489	ND	Pathogenic/Likely Pathogenic	
SIGILO386	*ATM*	11: 108284281	NM_000051.4(ATM):c.3802del (p.Glu1267_Val1268insTer)	nonsense	rs587779834	0.0000921	Pathogenic	
SIGILO387	*ATM*	11: 108284281	NM_000051.4(ATM):c.3802del (p.Glu1267_Val1268insTer)	nonsense	rs587779834	0.0000921	Pathogenic	
SIGILO388	*ATM*	11: 108294949-108294950	NM_000051.4(ATM):c.4801dup (p.Ser1601fs)	frameshift	rs2546942537	ND	Pathogenic	
SIGILO389	*ATM*	11: 108235825	NM_000051.4(ATM):c.487C>T (p.Gln163Ter)	nonsense	rs1591475608	ND	Pathogenic	
SIGILO390	*ATM*	11: 108259022	NM_000051.4(ATM):c.2413C>T (p.Arg805Ter)	nonsense	rs780619951	ND	Pathogenic	
SIGILO391	*ATM*	11: 108259022	NM_000051.4(ATM):c.2413C>T (p.Arg805Ter)	nonsense	rs780619951	ND	Pathogenic	
SIGILO392	*ATM*	11: 108325395	NM_000051.4(ATM):c.6658C>T (p.Gln2220Ter)	nonsense	rs1060501536	ND	Pathogenic	
SIGILO393	*ATM*	11: 108325425-108325426	NM_000051.4(ATM):c.6691_6692insCTTTT (p.Leu2231fs)	frameshift	rs2547203424	ND	Pathogenic	
SIGILO394	*ATM*	11: 108343338-108343347	NM_000051.4(ATM):c.8395_8404del (p.Phe2799fs)	frameshift	rs786202800	0.000013	Pathogenic	
SIGILO395	*ATM*	11: 108284281	NM_000051.4(ATM):c.3802del (p.Glu1267_Val1268insTer)	nonsense	rs587779834	0.0000921	Pathogenic	
SIGILO396	*ATM*	11: 108284281	NM_000051.4(ATM):c.3802del (p.Glu1267_Val1268insTer)	nonsense	rs587779834	0.0000921	Pathogenic	
SIGILO397	*ATM*	11: 108330281	NM_000051.4(ATM):c.7375C>G (p.Arg2459Gly)	missense	rs730881383	0.00004	Pathogenic	
SIGILO398	*CHEK2*	22: 28695869	NM_007194.4(CHEK2):c.1100del (p.Thr367fs)	frameshift	rs555607708	0.001748	Pathogenic	
SIGILO399	*CHEK2*	22: 28695869	NM_007194.4(CHEK2):c.1100del (p.Thr367fs)	frameshift	rs555607708	0.001748	Pathogenic	
SIGILO400	*CHEK2*	22: 28695869	NM_007194.4(CHEK2):c.1100del (p.Thr367fs)	frameshift	rs555607708	0.001748	Pathogenic	

Table S4. continued

ID	Gee	Chr:Pos (GRCh38)	Variant	Type	dbsnp	gnomad	Clinvar/ ACMG	Comment
SIGILO401	*CHEK2*	22: 29095824	NM_007194.4(CHEK2):c.1008+2T>G	splice donor	rs1555915295	0.0000021	Likely Pathogenic	
SIGILO402	*CHEK2*	22: 29095824	NM_007194.4(CHEK2):c.1008+2T>G	splice donor	rs1555915295	0.0000021	Likely Pathogenic	
SIGILO403	*CHEK2*	22: 29095824	NM_007194.4(CHEK2):c.1008+2T>G	splice donor	rs1555915295	0.0000021	Likely Pathogenic	
SIGILO404	*CHEK2*	22: 28696960	NM_007194.4(CHEK2):c.1036C>T (p.Arg346Cys)	missense	rs201206424	0.000007	Pathogenic	
SIGILO405	*CHEK2*	22: 28696957	NM_007194.4(CHEK2):c.1039G>A (p.Asp347Asn)	missense	rs28909980	ND	Likely Pathogenic	
SIGILO406	*CHEK2*	22: 28695869	NM_007194.4(CHEK2):c.1100del (p.Thr367fs)	frameshift	rs555607708	0.001748	Pathogenic	
SIGILO407	*CHEK2*	22: 28695869	NM_007194.4(CHEK2):c.1100del (p.Thr367fs)	frameshift	rs555607708	0.001748	Pathogenic	
SIGILO408	*CHEK2*	22: 28695869	NM_007194.4(CHEK2):c.1100del (p.Thr367fs)	frameshift	rs555607708	0.001748	Pathogenic	
SIGILO409	*CHEK2*	22: 28695869	NM_007194.4(CHEK2):c.1100del (p.Thr367fs)	frameshift	rs555607708	0.001748	Pathogenic	
SIGILO410	*CHEK2*	22: 28695841-28695842	NM_007194.4(CHEK2):c.1111_1127dup (p.Glu377fs)	frameshift	rs2052563898	ND	Pathogenic/Likely Pathogenic	
SIGILO411	*CHEK2*	22: 28695869	NM_007194.4(CHEK2):c.1100del (p.Thr367fs)	frameshift	rs555607708	0.001748	Pathogenic	
SIGILO412	*CHEK2*	22: 28725338	NM_007194.4(CHEK2):c.349A>G (p.Arg117Gly)	missense	rs28909982	0.0001948	Pathogenic/Likely Pathogenic	
SIGILO413	*CHEK2*	22: 28725338	NM_007194.4(CHEK2):c.349A>G (p.Arg117Gly)	missense	rs28909982	0.0001948	Pathogenic/Likely Pathogenic	
SIGILO414	*CHEK2*	22: 28725338	NM_007194.4(CHEK2):c.349A>G (p.Arg117Gly)	missense	rs28909982	0.0001948	Pathogenic/Likely Pathogenic	
SIGILO415	*CHEK2*	22: 28725338	NM_007194.4(CHEK2):c.349A>G (p.Arg117Gly)	missense	rs28909982	0.0001948	Pathogenic/Likely Pathogenic	
SIGILO416	*CHEK2*	22: 28725338	NM_007194.4(CHEK2):c.349A>G (p.Arg117Gly)	missense	rs28909982	0.0001948	Pathogenic/Likely Pathogenic	
SIGILO417	*CHEK2*	22: 28725099	NM_007194.4(CHEK2):c.470T>C (p.Ile157Thr)	missense	rs17879961	0.0026401	Pathogenic; Likely Pathogenic; Pathogenic, low penetrance; Established risk allele; Likely risk allele;	
SIGILO418	*CHEK2*	22: 28710005	NM_007194.4(CHEK2):c.846+1G>C	splice donor	rs864622149	0.000007	Pathogenic; Likely Pathogenic;	
SIGILO419	*CHEK2*	22: 28699909-28699910	NM_007194.4(CHEK2):c.936dup (p.Val313fs)	frameshift	rs1555915491	ND	Pathogenic; Likely Pathogenic;	
SIGILO420	*CHEK2*	22:29.092.888-29.095.926	NM_007194.3(CHEK2):c.909-?_1095+?del	Del exon 9-10		ND	Pathogenic	
SIGILO421	*CHEK2*	22: 28725254	NM_007194.4(CHEK2):c.433C>T (p.Arg145Trp)	missense	rs137853007	0.000047	Likely Pathogenic	
SIGILO422	*CHEK2*	22: 28695869	NM_007194.4(CHEK2):c.1100del (p.Thr367fs)	frameshift	rs555607708	0.001748	Pathogenic	
SIGILO423	*CHEK2*	22: 28710005	NM_007194.4(CHEK2):c.846+1G>A	splice donor	rs864622149	0.000007	Pathogenic	
SIGILO424	*CHEK2*	22: 28695737	NM_007194.4(CHEK2):c.1232G>A (p.Trp411Ter)	nonsense	rs371418985	0.0000029	Pathogenic/Likely Pathogenic	
SIGILO425	*CHEK2*	22: 28695869	NM_007194.4(CHEK2):c.1100del (p.Thr367fs)	frameshift	rs555607708	0.001748	Pathogenic	
SIGILO426	*CHEK2*	22: 28725338	NM_007194.4(CHEK2):c.349A>G (p.Arg117Gly)	missense	rs28909982	0.0001948	Pathogenic/Likely Pathogenic	
SIGILO427	*RAD51D*	17: 35106406	NM_002878.4(RAD51D):c.556C>T (p.Arg186Ter)	nonsense	rs387906843	0.000027	Pathogenic/Likely Pathogenic	
SIGILO428	*RAD51C*	17: 58703201	NM_058216.3(RAD51C):c.577C>T (p.Arg193Ter)	nonsense	rs200293302	ND	Pathogenic/Likely Pathogenic	
SIGILO429	*RAD51D*	17: 35101347	NM_002878.4(RAD51D):c.757C>T (p.Arg253Ter)	nonsense	rs137886232	ND	Pathogenic	

Table S4. continued

ID	Gee	Chr:Pos (GRCh38)	Variant	Type	dbsnp	gnomad	Clinvar/ ACMG	Comment
SIGILO430	*RAD51C*	17: 58709862	NM_058216.3(RAD51C):c.709C>T (p.Arg237Ter)	nonsense	rs770637624	0.0000057	Pathogenic	
SIGILO431	*RAD51C*	17: 58720795-58720804	NM_058216.3(RAD51C):c.890_899del (p.Leu297fs)	frameshift	rs1555602141	0	Pathogenic	
SIGILO432	*RAD51C*	17: 58720795-58720804	NM_058216.3(RAD51C):c.890_899del (p.Leu297fs)	frameshift	rs1555602141	0	Pathogenic	
SIGILO433	*RAD51C*	17: 58695189	NM_058216.3(RAD51C):c.404G>A (p.Cys135Tyr)	missense	rs767796996	0.000003	Pathogenic/Likely Pathogenic	
SIGILO434	*RAD51C*	17: 58695189	NM_058216.3(RAD51C):c.404G>A (p.Cys135Tyr)	missense	rs767796996	0.000003	Pathogenic/Likely Pathogenic	
SIGILO435	*RAD51C*	17: 58694964-58694965	NM_058216.3(RAD51C):c.181_182del (p.Leu61fs)	frameshift	rs786203945	0.000007	Pathogenic	
SIGILO436	*RAD51D*	17: 35103298	NM_002878.4(RAD51D):c.694C>T (p.Arg232Ter)	nonsense	rs587780104	0.000034	Pathogenic/Likely Pathogenic	
SIGILO437	*RAD51C*	17: 58720795-58720804	NM_058216.3(RAD51C):c.890_899del (p.Leu297fs)	frameshift	rs1555602141	0	Pathogenic	
SIGILO438	*RAD51C*	17: 58694964-58694965	NM_058216.3(RAD51C):c.181_182del (p.Leu61fs)	frameshift	rs786203945	0.000007	Pathogenic	
SIGILO439	*RAD51C*	17: 58709862	NM_058216.3(RAD51C):c.709C>T (p.Arg237Ter)	nonsense	rs770637624	0.0000057	Pathogenic	
SIGILO440	*RAD51C*	17: 58695189	NM_058216.3(RAD51C):c.404G>A (p.Cys135Tyr)	missense	rs767796996	0.000003	Pathogenic/Likely Pathogenic	
SIGILO441	*RAD51C*	17: 58696692-58696858	NM_058216.3(RAD51C):c.405_571del	splice acceptor	rs2143742992	ND	Pathogenic	
SIGILO442	*RAD51C*	17: 58720795-58720804	NM_058216.3(RAD51C):c.890_899del (p.Leu297fs)	frameshift	rs1555602141	0	Pathogenic	
SIGILO443	*RAD50*	5: 132589653-132589654	NM_005732.4(RAD50):c.1270_1271del (p.Leu424fs)	frameshift	rs587781327	0.0000123	Pathogenic	
SIGILO444	*RAD51C*	17: 58720795-58720804	NM_058216.3(RAD51C):c.890_899del (p.Leu297fs)	frameshift	rs1555602141	0	Pathogenic	
SIGILO445	*RAD51D*	17: 35103298	NM_002878.4(RAD51D):c.694C>T (p.Arg232Ter)	nonsense	rs587780104	0.000034	Pathogenic/Likely Pathogenic	
SIGILO446	*RAD51C*	17: 58720795-58720804	NM_058216.3(RAD51C):c.890_899del (p.Leu297fs)	frameshift	rs1555602141	0	Pathogenic	
SIGILO447	*RAD51C*	17: 58695189	NM_058216.3(RAD51C):c.404G>A (p.Cys135Tyr)	missense	rs767796996	0.000003	Pathogenic/Likely Pathogenic	
SIGILO448	*RAD51C*	17: 58695006-58695007	NM_058216.3(RAD51C):c.224_225dup (p.Ala76fs)	frameshift	rs1598455598	0.0000007	Pathogenic/Likely Pathogenic	
SIGILO449	*FANCL*	:58161533-58161535	NM_018062.4(FANCL):c.1007_1009del (p.Ile336_Cys337delinsSer)	inframe del	rs747253294	0.0005472	Likely pathogenic	
SIGILO450	*FANCL*	2: 58160148-58160149	NM_018062.4(FANCL):c.1051_1052del (p.Ser351fs)	frameshift	rs750871999	0.000040	Likely Pathogenic	
SIGILO451	*FANCM*	14:45155424-45155425 (GRCh38.p14)	NM_020937.4(FANCM):c.1362_1363insCAAAGTTAAA(p.Glu455Glnfs*3)	frameshift	rs774390409	ND	Likely Pathogenic	
SIGILO452	*FANCI*	15: 89285201	NM_001113378.2(FANCI):c.1804C>T (p.Arg602Ter)	nonsense	rs1432325198	0.0000078	Pathogenic/Likely Pathogenic	
SIGILO453	*FANCM*	14: 45167133	NM_020937.4(FANCM):c.1972C>T (p.Arg658Ter)	nonsense	rs368728266	0.0000900	Pathogenic/Likely Pathogenic	
SIGILO454	*FANCD2*	3: 10081465	NM_001018115.3(FANCD2):c.3224+1G>C	splice donor	rs1468320596	ND	Likely Pathogenic	
SIGILO455	*MLH1*	3: 37025635	NM_000249.4(MLH1):c.1039-2A>G	splice acceptor	rs267607815	ND	Likely Pathogenic	

Table S4. continued

ID	Gee	Chr:Pos (GRCh38)	Variant	Type	dbsnp	gnomad	Clinvar/ ACMG	Comment
SIGILO456	*MLH1*	3: 37020312	NM_000249.4(MLH1):c.887T>G (p.Leu296Ter)	nonsense	rs63750547	0.000002	Pathogenic	
SIGILO457	*MSH2*	2: 47429881	NM_000251.3(MSH2):c.1216C>T (p.Arg406Ter)	nonsense	rs63751108	0.000004	Pathogenic	
SIGILO458	*PMS2*	7: 5999116	NM_000535.7(PMS2):c.697C>T (p.Gln233Ter)	nonsense	rs587779343	ND	Pathogenic	
SIGILO459	*PMS2*	7: 5982903	NM_000535.7(PMS2):c.2095G>C (p.Asp699His)	missense	rs587781317	0.000002	Pathogenic	
SIGILO460	*PMS2*	7: 5999116	NM_000535.7(PMS2):c.697C>T (p.Gln233Ter)	nonsense	rs587779343	ND	Pathogenic	
SIGILO461	*MSH6*	2: 47800840	NM_000179.3(MSH6):c.2857G>T (p.Glu953Ter)	nonsense	rs753034685	0	Pathogenic	
SIGILO462	*MSH6*	2: 47806279-47806281	NM_000179.3(MSH6):c.3724_3726del (p.Arg1242del)	inframe deletion	rs63749942	0.0000029	Likely Pathogenic	
SIGILO463	*MSH6*	2: 47799501-47799502	NM_000179.3(MSH6):c.1519dup (p.Arg507fs)	frameshift	rs876658881	0.0000007	Pathogenic	
SIGILO464	*MSH6*	2: 47798875	NM_000179.3(MSH6):c.892C>T (p.Arg298Ter)	nonsense	rs146816935	0.000007	Pathogenic	
SIGILO465	*PMS2*	7: 5978687-5978689	NM_000535.6(PMS2):c.2182_2184delinsG (p.Thr728Alafs)	frameshift	rs1554294508	ND	Pathogenic; Likely Pathogenic	
SIGILO466	*MSH2*	2: 47429881	NM_000251.3(MSH2):c.1216C>T (p.Arg406Ter)	nonsense	rs63751108	0.000004	Pathogenic	
SIGILO467	*MUTYH*	1: 45331556	NM_001048174.2(MUTYH):c.1103G>A (p.Gly368Asp)	missense	rs36053993	0.003349	Pathogenic/Likely Pathogenic	
SIGILO468	*MUTYH*	1: 45331556	NM_001048174.2(MUTYH):c.1103G>A (p.Gly368Asp)	missense	rs36053993	0.003349	Pathogenic/Likely Pathogenic	
SIGILO469	*MUTYH*	1: 45332443	NM_001048174.2(MUTYH):c.652G>T (p.Val218Phe)	missense	rs587780749	ND	Pathogenic/Likely Pathogenic	
SIGILO470	*BRIP1*	17: 61716051	NM_032043.3(BRIP1):c.2392C>T (p.Arg798Ter)	nonsense	rs137852986	0.0002911	Pathogenic	
SIGILO471	*BRIP1*	17: 61684141	NM_032043.3(BRIP1):c.2906-1G>A	splice acceptor	rs773639563	0.0000021	Likely Pathogenic	
SIGILO472	*BRIP1*	17: 61716051	NM_032043.3(BRIP1):c.2392C>T (p.Arg798Ter)	nonsense	rs137852986	0.0002911	Pathogenic	
SIGILO473	*BRIP1*	17: 61716051	NM_032043.3(BRIP1):c.2392C>T (p.Arg798Ter)	nonsense	rs137852986	0.0002911	Pathogenic	
SIGILO474	*BRIP1*	17: 61716051	NM_032043.3(BRIP1):c.2392C>T (p.Arg798Ter)	nonsense	rs137852986	0.0002911	Pathogenic	
SIGILO475	*BRIP1*	17: 61861440-61861443	NM_032043.3(BRIP1):c.93+4_93+7del	splice donor	rs1224034842	0.0000014	Likely Pathogenic	
SIGILO476	*BRIP1*	17:59.924.461-59.926.617	Deletion exons 5-6 (2.1kb)	deletion			Pathogenic	
SIGILO477	*BRIP1*	17: 61776564	NM_032043.3(BRIP1):c.1936-2A>C	splice acceptor	rs878855143	ND	Pathogenic	
SIGILO478	*XRCC2*	7: 152649135-152649138	NM_005431.2(XRCC2):c.347_350del (p.Phe116fs)	frameshift	rs764640893	0.000027	Pathogenic	
SIGILO479	*BARD1*	2: 214797099-214797100	NM_000465.4(BARD1):c.176_177del (p.Glu59fs)	frameshift	rs1057517589	0.000007	Pathogenic/Likely Pathogenic	
SIGILO480	*BARD1*	2: 214780669	NM_000465.4(BARD1):c.1205C>A (p.Ser402Ter)	nonsense	rs796666047	0.000007	Pathogenic/Likely Pathogenic	
SIGILO481	*BARD1*	2: 214797099-214797100	NM_000465.4(BARD1):c.176_177del (p.Glu59fs)"	frameshift	rs1057517589	0.000007	Pathogenic/Likely Pathogenic	
SIGILO482	*PTEN*	10: 87961033	NM_000314.8(PTEN):c.942del (p.Glu314fs)	frameshift	ND	ND	Pathogenic	
SIGILO483	*PTEN*	10: 89653867	NM_000314.8(PTEN):c.164+1G>A	splice donor	rs1554893835	ND	Pathogenic	
SIGILO484	*NF1*	17: 31327718	NM_001042492.3(NF1):c.5488C>T (p.Arg1830Cys)	missense	rs797045139	0.0000021	Pathogenic	
SIGILO485	*NBN*	8: 89947893	NM_002485.5(NBN):c.1846-1G>T	splice acceptor	rs61753717	0	Pathogenic	
SIGILO486	*MRE11*	11: 94470564-94470565	NM_005591.4(MRE11):c.923dup (p.Met309fs)	frameshift	rs587781828	0	Pathogenic/Likely Pathogenic	
SIGILO487	*RECQL*	12: 21490301	NM_002907.4(RECQL):c.292G>T (p.Glu98Ter)	nonsense	rs1010112210	ND	Likely Pathogenic	
SIGILO488	*CDH1*	16: 68810224	NM_004360.5(CDH1):c.715G>A (p.Gly239Arg)	missense	rs587780537	0.0000014	Pathogenic	

Table S4. continued

ID	Gee	Chr:Pos (GRCh38)	Variant	Type	dbsnp	gnomad	Clinvar/ ACMG	Comment
SIGILO489	*NTHL1*	16: 2047708	NM_002528.7(NTHL1):c.115+1G>A	splice donor	rs749908882	0.000020	Pathogenic/Likely Pathogenic	
SIGILO490	*NF1*	17: 31327718	NM_001042492.3(NF1):c.5488C>T (p.Arg1830Cys)	missense	rs797045139	0.0000021	Pathogenic	
SIGILO491	*MUTYH*	1: 45332443	NM_001048174.2(MUTYH):c.652G>T (p.Val218Phe)	missense	rs587780749	ND	Pathogenic/Likely Pathogenic	
SIGILO492	*BRIP1*	17: 61849152	NM_032043.3(BRIP1):c.484C>T (p.Arg162Ter)	nonsense	rs747604569	0.000007	Pathogenic/Likely Pathogenic	
SIGILO493	*MUTYH*	1:45797228	NM_001128425.2(MUTYH):c.1187G>A (p.Gly396Asp)	missense	rs36053993	0.003349	Pathogenic/Likely Pathogenic	
SIGILO494	MINAS	17: 43067606 ; 7: 5989822-5989825	NM_007294.4(BRCA1):c.5074+2T>C/ NM_000535.7(PMS2):c.1119_1122del (p.Gln374fs)	splice donor/ frameshift	rs80358089/ rs757679199	ND	Pathogenic (BRCA1)/ Pathogenic/Likely Pathogenic (PMS2)	
SIGILO495	MINAS	16: 236262732; 2: 28734401	NM_024675.4(PALB2):c.2711G>A (p.Trp904Ter); NM_007194.4(CHEK2):c.319+2T>A	nonsense; splice donor	rs1060502726; rs587782401	0.0000021; 0.000087	Pathogenic; Pathogenic/Likely Pathogenic	
SIGILO496	MINAS	22: 28734637; 1: 45332803	NM_007194.4(CHEK2):c.85C>T (p.Gln29Ter); NM_001048174.2(MUTYH):c.452A>G (p.Tyr151Cys)	nonsense; missense	rs761494650; rs34612342	0.0000057; 0.0019586 (	both: Pathogenic/Likely Pathogenic	
SIGILO497	MINAS	13-32914872; 17: 58709862	NM_000059.4(BRCA2): c.6381_6382insTT p.(Lys2128Leufs*10) NM_058216.3(RAD51C):c.709C>T (p.Arg237Ter)	frameshift; nonsense	ND; rs767796996	ND; 0.0000057	Likely Pathogenic; Pathogenic	
SIGILO498	MINAS	1: 45331556; 16: 2047708	NM_001048174.2(MUTYH):c.1103G>A (p.Gly368Asp); NM_002528.7(NTHL1):c.115+1G>A	missense; splice donor	rs36053993; rs749908882	0.003349 ; 0.000020	both:Pathogenic/Likely Pathogenic	
SIGILO499	MINAS	1:45797228; 17: 61799235-61799236	NM_001128425.2(MUTYH):c.1187G>A (p.Gly396Asp) ; NM_032043.3(BRIP1):c.1201_1204dup (p.Ala402fs)	missense; frameshift	rs36053993; rs730881647	0.003349 ; ND	Pathogenic/Likely pathogenic; Pathogenic	
SIGILO500	MINAS	17: 7673821 ; 13: 32319075	NM_000546.6(TP53):c.799C>T (p.Arg267Trp); NM_000059.4(BRCA2):c.68-2A>G	missense; splice acceptor	rs55832599; rs769152395	0.000003 ; 0.000003	Pathogenic/Likely Pathogenic; Likely Pathogenic	
SIGILO501	MINAS	1: 45332490; 14: 75515731 ; 17-63554730 GCTACTCATGGTGA GGGAGCTC>G	NM_001048174.2(MUTYH):c.607-2A>C; NM_001040108.2(MLH3):c.628C>T (p.Arg210Ter); NM_004655.4(AXIN2):c.-12_8del (p.Met1fs) - Oligodontia-Colorectal Cancer Syndrome (OMIM 608615, ORPHA 300576)	splice acceptor; nonsense; frameshift initiator codon variant	rs1361763237; rs770668994; rs768265778	ND; 0.0000521 ; 0.000013	Likely Pathogenic; Likely Pathogenic; Pathogenic (**patient with oligodontia)	
SIGILO502	MINAS	22: 28725338; 13: 32341071	NM_007194.4(CHEK2):c.349A>G (p.Arg117Gly); NM_000059.4(BRCA2):c.6717del (p.Glu2239fs)	missense; frameshift	rs28909982; rs1593909597	0.0001948 ; ND	Pathogenic/Likely Pathogenic; Pathogenic	
SIGILO503	MINAS	22: 29095824; 11: 94478789-94478790	NM_007194.4(CHEK2):c.1008+2T>G; NM_005591.4(MRE11):c.489_490del (p.Asp163_Ile164insTer)	splice donor; frameshift	rs1555915295; rs2496555781	0.0000021; ND	Likely Pathogenic; Pathogenic	

Table S5. Distribution of histological grade according to germline gene

Germline gene	Grade I	Grade II	Grade III	Total
ATM	3	18	9	30
BARD1	1	1	1	3
BRCA1	0	48	108	156
BRCA2	4	47	55	106
BRIP1	2	2	5	9
CDH1	0	0	1	1
CHEK2	5	16	7	28
FANCD2	1	0	0	1
FANCI	0	0	1	1
FANCL	1	0	0	1
FANCM	0	0	2	2
MLH1	1	1	0	2
MRE11	0	0	1	1
MSH2	0	1	1	2
MSH6	0	2	2	4
MUTYH	1	1	2	4
NBN	1	0	0	1
NF1	0	1	0	1
NTHL1	0	1	0	1
PALB2	4	15	11	30
PMS2	0	2	2	4
PTEN	0	1	1	2

Table S5. continued

Germline gene	Grade I	Grade II	Grade III	Total
RAD50	0	0	1	1
RAD51C	3	4	9	16
RAD51D	0	2	3	5
TP53, other variants	2	4	5	11
TP53 p.R337H	1	26	22	49
XRCC2	0	0	1	1
**Total**	**32**	**196**	**254**	**482**

Data are presented as numbers of tumors. Histological grade was assessed using the Scarff–Bloom–Richardson (SBR) grading system and was available for 482 tumors. The distribution of SBR grade differed significantly across germline genes (Fisher's exact test with Monte Carlo simulation, $p < 0.001$). *TP53* p.R337H denotes the Brazilian founder variant *TP53* c.1010G>A (p.R337H).

Supplementary Table S6. Genetic composition within each SBR grade

Germline gene or gene category	Grade I (%)	Grade II (%)	Grade III (%)
BRCA1	0.0	24.9	43.2
BRCA2	13.3	24.4	22.0
TP53 p.R337H	3.3	13.5	8.8
CHEK2	16.7	8.3	2.8
PALB2	13.3	7.8	4.4
ATM	10.0	9.3	3.6
RAD51C	10.0	2.1	3.6
Other *TP53* variants	6.7	2.1	2.0
Other genes combined	26.7	7.6	10.6
**Total**	**100.0**	**100.0**	**100.0**

Histological grade was classified according to the Scarff–Bloom–Richardson (SBR) system. Percentages were calculated within each SBR grade and therefore sum to approximately 100% within each column; minor discrepancies may occur because of rounding. Only tumors with available SBR grade data were included (n = 482). Detailed gene-level absolute frequencies are provided in Supplementary Table S5. *TP53* p.R337H denotes the Brazilian founder variant *TP53* c.1010G>A (p.R337H).

Supplementary Table S7. Distribution of nuclear grade according to germline gene

Germline gene or category	Grade I	Grade II	Grade III	Total
ATM	2	10	17	29
BARD1	0	1	1	2
BRCA1	0	27	126	153
BRCA2	1	31	75	107
BRIP1	0	3	6	9
CDH1	0	0	1	1
CHEK2	2	11	14	27
MINAS	1	4	4	9
FANCD2	1	0	0	1
FANCI	0	0	1	1
FANCL	0	1	0	1
FANCM	0	0	2	2
MLH1	0	2	0	2
MRE11	0	0	1	1
MSH2	0	1	1	2
MSH6	0	2	2	4
MUTYH	1	0	3	4
NBN	1	0	0	1
NF1	0	0	1	1
NTHL1	0	0	1	1
PALB2	3	9	18	30
PMS2	0	1	3	4
PTEN	0	1	1	2
RAD50	0	0	1	1
RAD51C	2	3	11	16

Supplementary Table S7.continued

Germline gene or category	Grade I	Grade II	Grade III	Total
RAD51D	0	1	4	5
Other *TP53* variants	1	4	7	12
TP53 p.R337H	0	13	35	48
XRCC2	0	0	1	1
**Total**	**15**	**122**	**332**	**469**

Data are presented as numbers of tumors. Nuclear grade was available for 469 tumors. The distribution of nuclear grade differed significantly across germline genes (Fisher's exact test with Monte Carlo simulation, $p < 0.001$). *TP53* p.R337H denotes the Brazilian founder variant *TP53* c.1010G>A (p.R337H). MINAS=multilocus inherited neoplasia allele syndrome.

Supplementary Table S8. Distribution of nuclear grade within selected germline genes

Germline gene or variant category	Grade I (%)	Grade II (%)	Grade III (%)
ATM	6.9	34.5	58.6
BRCA1	0.0	17.6	82.4
BRCA2	0.9	29.0	70.1
CHEK2	7.4	40.7	51.9
PALB2	10.0	30.0	60.0
RAD51C	12.5	18.8	68.8
Other *TP53* variants	8.3	33.3	58.3
TP53 p.R337H	0.0	27.1	72.9

Percentages were calculated within each germline gene or variant category and therefore sum to approximately 100% across each row; minor discrepancies may occur because of rounding. The table presents selected genes and variant categories with sufficient informative cases. Absolute frequencies for all genes are provided in Supplementary Table S7. Results for categories with limited sample sizes should be interpreted cautiously. *TP53* p.R337H denotes the Brazilian founder variant *TP53* c.1010G>A (p.R337H).

Supplementary Table S9. Gene-specific associations with high tumor-infiltrating lymphocyte levels

Germline gene or variant category	Informative cases, n	High TILs, n (%)	OR	95% CI	*p* value	FDR-adjusted *p* value
BRCA1	134	67 (50.0)	2.63	1.66–4.16	0.000013	0.000091
ATM	25	4 (16.0)	0.33	0.08–1.01	0.0496	0.174
TP53 p.R337H	36	8 (22.2)	0.50	0.19–1.16	0.101	0.235
BRCA2	85	25 (29.4)	0.72	0.41–1.23	0.248	0.434
PALB2	25	7 (28.0)	0.70	0.24–1.82	0.520	0.728
RAD51C	14	4 (28.6)	0.73	0.16–2.59	0.778	0.908
CHEK2	25	9 (36.0)	1.04	0.39–2.58	1.000	1.000

High TIL levels were defined as moderate or intense stromal lymphocytic infiltration (≥ 10%), whereas low TIL levels were defined as discrete infiltration (< 10%). For each gene or variant category, the odds of high TIL levels among carriers were compared with those among all other patients with evaluable TIL data. Odds ratios and 95% confidence intervals were estimated from gene-specific 2×2 analyses. P values were adjusted for multiple comparisons using the Benjamini–Hochberg false discovery rate method. Only genes or variant categories with at least ten informative cases are shown. *TP53* p.R337H denotes the Brazilian founder variant *TP53* c.1010G>A (p.R337H). TILs=tumor-infiltrating lymphocytes; OR=odds ratio; CI=confidence interval; FDR=false discovery rate.

Supplementary Table S10. Distribution of tumor molecular phenotypes

**Tumor molecular phenotype**	**n**	**Proportion (%)**
Triple-negative	160	31.8
Luminal B	123	24.4
Luminal B HER2-low	93	18.5
Luminal B HER2-positive	62	12.3
Luminal A	32	6.4
HER2-positive non-luminal	14	2.8
Triple-negative HER2-low	10	2.0
Basal-like	5	1.0
Luminal A HER2-low	4	0.8
**Total**	**503**	**100.0**

Data are presented as n (%). Tumor molecular phenotype was available for all 503 patients. HER2=human epidermal growth factor receptor 2.

Supplementary Table S11. Associations between germline genetic groups and tumor molecular phenotypes

Germline genetic group	Tumor molecular phenotype	Direction of association	Standardised residual (z)
BRCA1	Triple-negative	Enrichment	+10.59
BRCA1	Luminal B HER2-low	Depletion	−5.53
BRCA1	Luminal B HER2-positive	Depletion	−4.55
BRCA1	Luminal A	Depletion	−2.80
TP53	Luminal B HER2-positive	Enrichment	+4.36
TP53	Triple-negative	Depletion	−4.25
ATM	Luminal B HER2-positive	Enrichment	+3.25
ATM	Triple-negative	Depletion	−4.06
CHEK2	Luminal B HER2-low	Enrichment	+3.27
CHEK2	Triple-negative	Depletion	−2.97
BRCA2	Luminal B HER2-low	Enrichment	+2.47
BRCA2	Luminal B HER2-positive	Depletion	−2.12
PALB2	Luminal B HER2-low	Enrichment	+2.16
RAD family	Luminal B	Depletion	−2.79
MMR genes	Luminal A HER2-low	Enrichment	+12.84
MMR genes	Triple-negative	Depletion	−2.39

Associations between germline genetic groups and tumor molecular phenotypes were assessed using Fisher's exact test with Monte Carlo simulation. The global association was statistically significant ($p < 0.001$), with a moderate effect size (Cramér's V = 0.312). Only gene group–phenotype combinations with absolute standardized residuals $|z| \geq 2$ are shown. Positive residuals indicate enrichment and negative residuals indicate depletion relative to expected frequencies. Standardized residuals are descriptive and should not be interpreted as independently tested associations. RAD family includes *RAD51C, RAD51D,* and *XRCC2*. MMR genes include *MLH1, MSH2, MSH6,* and *PMS2*. HER2=human epidermal growth factor receptor 2.

Survival Outcomes

Supplementary Kaplan–Meier analyses of local recurrence-free survival, progression-free survival, and overall survival are presented in Figures S2, S3, and S4, respectively. Definitions of the survival endpoints, censoring rules, numbers of events, and corresponding exploratory log-rank results are provided in the figure legends.

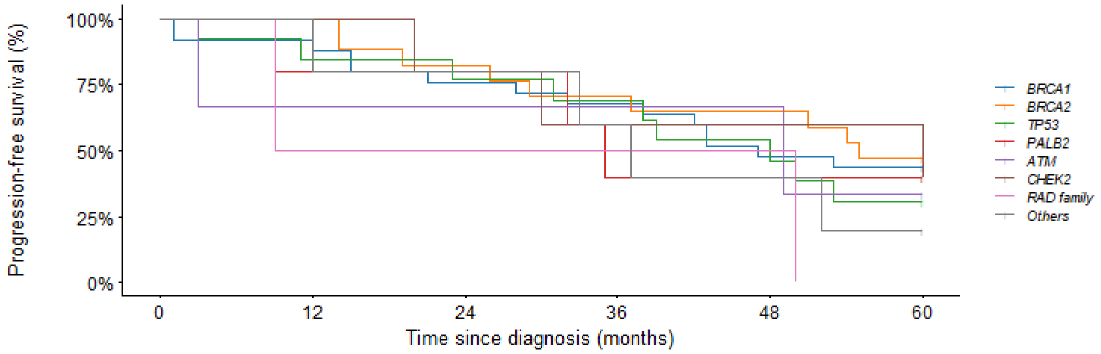

Number at risk

	0	12	24	36	48	60
BRCA1	25	23	19	17	12	11
BRCA2	17	17	14	12	11	8
TP53	13	11	10	9	7	4
PALB2	5	4	4	2	2	2
ATM	3	2	2	2	2	1
CHEK2	5	5	4	3	3	3
RAD family	2	1	1	1	1	0
Others	5	5	4	3	2	1

Time since diagnosis (months)

Figure S2. Kaplan–Meier estimates of local recurrence-free survival according to germline genetic group. Local recurrence-free survival was analysed in 500 patients, with 20 local recurrence events recorded within 60 months. Survival distributions were compared using the log-rank test (χ^2 = 8.21, df=8; p = 0.413). Follow-up was administratively censored at 60 months. Shaded areas represent 95% confidence intervals. Tick marks indicate censored observations.

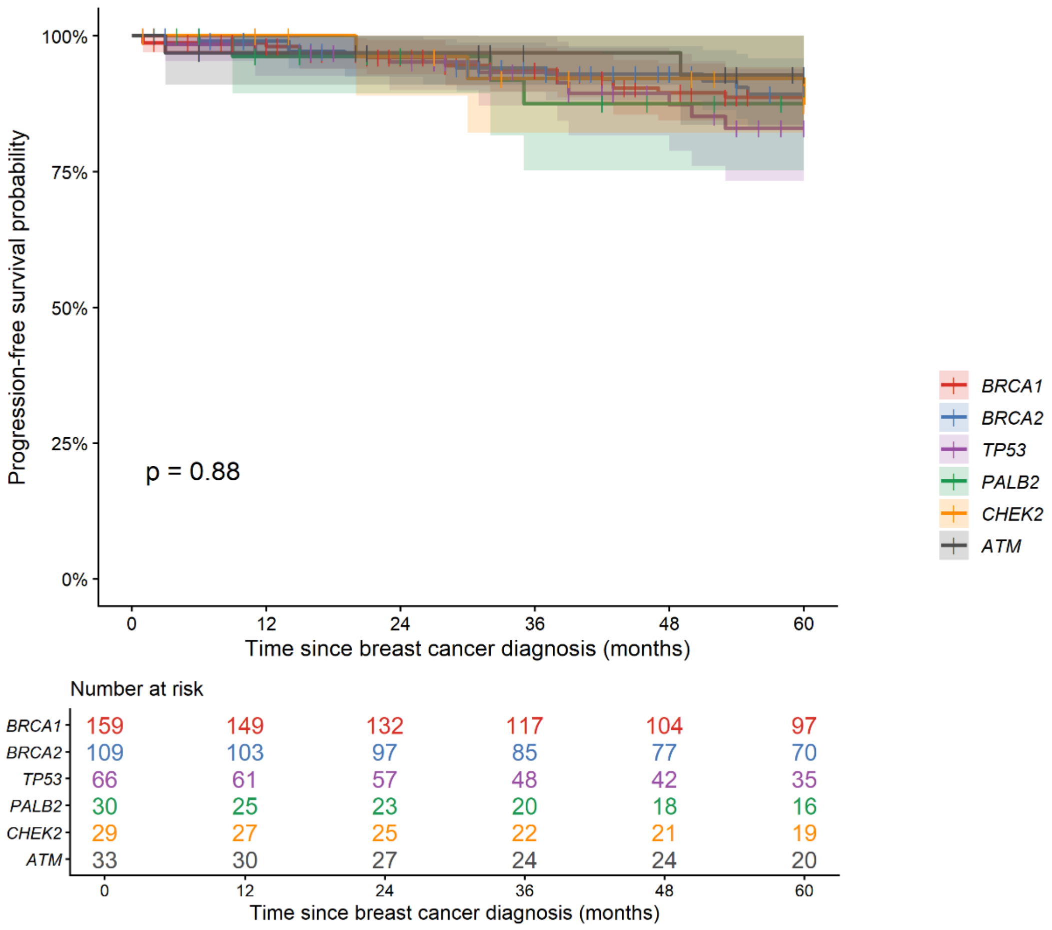

Figure S3. **Progression-free survival according to the main germline susceptibility genes.** Kaplan–Meier curves show 60-month progression-free survival for patients carrying P/LP germline variants in *BRCA1, BRCA2, TP53, PALB2, CHEK2,* or *ATM*. Progression-free survival was defined as the interval from breast cancer diagnosis to the first documented disease progression, including local recurrence, or death from any cause. Shaded areas represent 95% confidence intervals, vertical marks indicate censored observations, and the table shows the number of patients at risk at each time point. Follow-up was administratively censored at 60 months. No statistically significant difference was detected across the six genes in the exploratory log-rank analysis (p = 0.88).

Table S12. PFS at 60 months by genetic group

Group	n	Events	PFS at 60 months
BRCA1	159	15	88.6%
BRCA2	109	10	89.2%
TP53	66	9	83.0%
ATM	33	2	92.8%
Other rare genes	30	5	76.7%
PALB2	30	3	87.5%
CHEK2	29	3	87.3%
RAD group	22	2	86.4%
MMR group	12	0	100%
MINAS	10	1	83.3%
MRN complex	3	0	100%

Overall PFS at 60 months: 87.7% (95% CI: 84.5–91.0%)

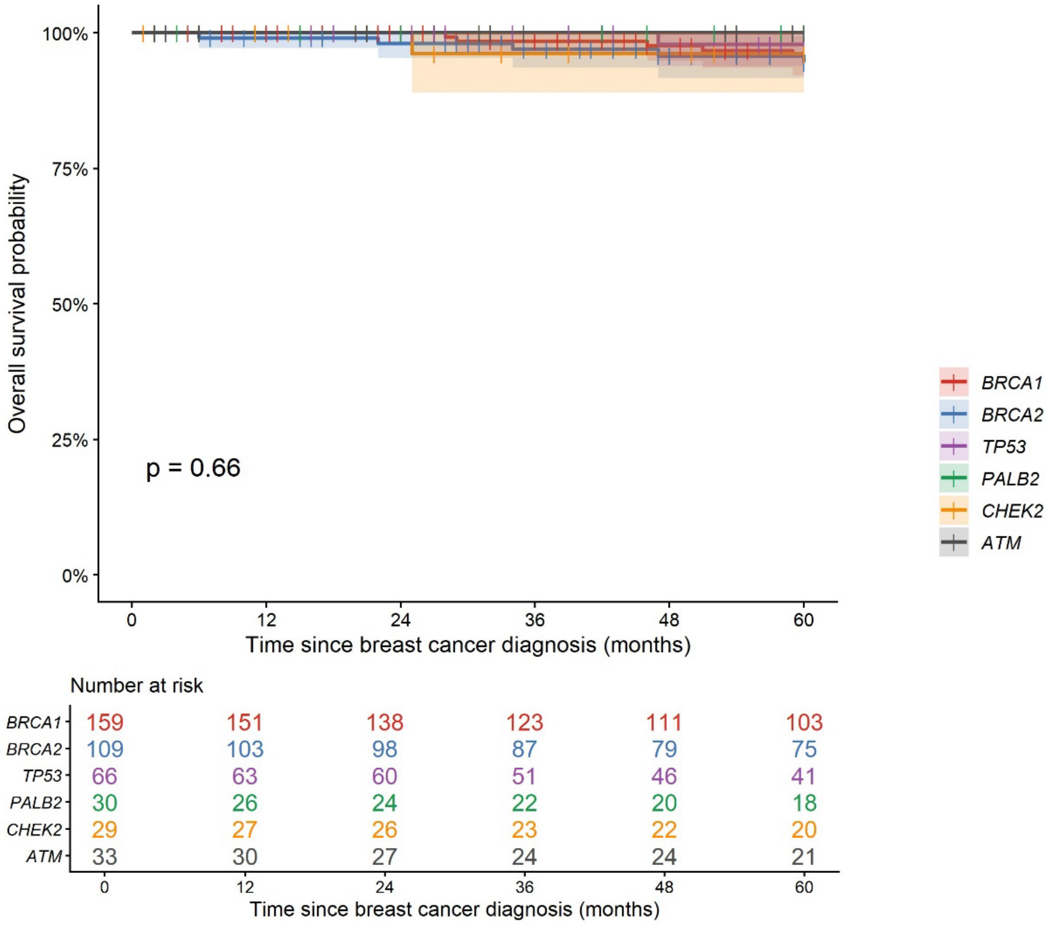

Figure S4. Overall survival according to the main germline susceptibility genes. Kaplan–Meier curves show 60-month overall survival for patients carrying P/LP germline variants in *BRCA1, BRCA2, TP53, PALB2, CHEK2,* or *ATM*. Overall survival was defined as the interval from breast cancer diagnosis to death from any cause. Patients alive at their last documented assessment, including those subsequently lost to follow-up, were censored at that time, and follow-up was administratively censored at 60 months. Shaded areas represent 95% confidence intervals, vertical marks indicate censored observations, and the table shows the number of patients at risk. No statistically significant difference was detected across the six genes in the exploratory log-rank analysis ($p = 0.664$).

Table S13- Genes included in the common 100-gene multigene NGS panel used in 333 patients.

Gene	Gene	Gene	Gene
AIP	*CTNNA1*	*MSH2*	*RB1*
AKT1	*DICER1*	*MSH3*	*RECQL*
ALK	*DIS3L2*	*MSH6*	*RECQL4*
APC	*EGFR*	*MUTYH*	*RET*
ATM	*EPCAM*	*NBN*	*RNF43*
ATP4A	*FANCA*	*NF1*	*RPS20*
ATR	*FANCC*	*NF2*	*RUNX1*
AXIN2	*FANCL*	*NTHL1*	*SDHA*
BAP1	*FANCM*	*PALB2*	*SDHAF2*
BARD1	*FH*	*PDGFRA*	*SDHB*
BLM	*FLCN*	*PHOX2B*	*SDHC*
BMPR1A	*GATA2*	*PIK3CA*	*SDHD*
BRCA1	*GPC3*	*PMS2*	*SMAD4*
BRCA2	*GREM1*	*POLD1*	*SMARCA4*
BRIP1	*HOXB13*	*POLE*	*SMARCB1*
CDC73	*IPMK*	*POT1*	*SMARCE1*
CDH1	*KIT*	*PRKAR1A*	*STK11*
CDK12	*LZTR1*	*PTCH1*	*SUFU*
CDK4	*MAX*	*PTEN*	*TERT*
CDKN1B	*MBD4*	*RABL3*	*TMEM127*
CDKN1C	*MEN1*	*RAD50*	*TP53*
CDKN2A	*MET*	*RAD51*	*TSC1*
CEBPA	*MITF*	*RAD51B*	*TSC2*
CHEK1	*MLH1*	*RAD51C*	*VHL*
CHEK2	*MRE11*	*RAD51D*	*XRCC2*
